# Chromatin states shape insertion profiles of the piggyBac, Tol2 and Sleeping Beauty transposons and murine leukemia virus

**DOI:** 10.1038/srep43613

**Published:** 2017-03-02

**Authors:** Junko Yoshida, Keiko Akagi, Ryo Misawa, Chikara Kokubu, Junji Takeda, Kyoji Horie

**Affiliations:** 1 Department of Physiology II, Nara Medical University, Kashihara, Nara 634-8521, Japan; 2 Department of Genome Biology, Graduate School of Medicine, Osaka University, Suita, Osaka 565-0871, Japan; 3 Comprehensive Cancer Center, Ohio State University, Columbus, Ohio 43210, USA; 4 Precursory Research for Embryonic Science and Technology, Japan Science and Technology Agency, Kawaguchi, Saitama 332-0012, Japan

## Abstract

DNA transposons and retroviruses are versatile tools in functional genomics and gene therapy. To facilitate their application, we conducted a genome-wide insertion site profiling of the piggyBac (PB), Tol2 and Sleeping Beauty (SB) transposons and the murine leukemia virus (MLV) in mouse embryonic stem cells (ESCs). PB and MLV preferred highly expressed genes, whereas Tol2 and SB preferred weakly expressed genes. However, correlations with DNase I hypersensitive sites were different for all vectors, indicating that chromatin accessibility is not the sole determinant. Therefore, we analysed various chromatin states. PB and MLV highly correlated with Cohesin, Mediator and ESC-specific transcription factors. Notably, CTCF sites were correlated with PB but not with MLV, suggesting MLV prefers smaller promoter–enhancer loops, whereas PB insertion encompasses larger chromatin loops termed topologically associating domains. Tol2 also correlated with Cohesin and CTCF. However, correlations with ESC-specific transcription factors were weaker, suggesting that Tol2 prefers transcriptionally weak chromatin loops. Consistently, Tol2 insertions were associated with bivalent histone modifications characteristic of silent and inducible loci. SB showed minimum preference to all chromatin states, suggesting the least adverse effect on adjacent genes. These results will be useful for vector selection for various applications.

DNA transposons and retroviruses have been widely used as invaluable tools in various fields of life science, including gene therapy^1^, cancer gene discovery^2^,^3^,^4^, and insertional mutagenesis^5^,^6^. Retroviruses have been the most popular vector for application in mammalian cells for the last few decades because of their high efficiency of infection. Compared to retroviruses, the use of DNA transposons has been hampered for many years because most DNA transposons are inactivated during evolution and active DNA transposons have not been available. However, resurrection of the Sleeping Beauty (SB) transposon from the salmonid fish genome^7^ ignited the development of a series of active transposons. Currently, there are several available DNA transposon vectors such as piggyBac (PB) from cabbage looper moth, Trichoplusia ni^8^ and Tol2 from medaka fish^9^.

We have extensively utilized DNA transposons and murine leukemia virus (MLV) for functional genomics and gene transfer. We developed a method of transposon-tagged germline mutagenesis in mice using the SB transposon and generated more than 300 mutant mouse lines^10^,^11^,^12^. We also utilized MLV, Tol2 and PB for insertional mutagenesis of mouse embryonic stem cells (ESCs) and generated more than 1,000 mutant cell lines^5^. During these experiments, we observed that the same genes were repeatedly inserted by the same vectors, indicating the substantial bias of vector insertion sites. From this, we realized that information regarding vector insertion preference is necessary when choosing an appropriate vector in various experimental settings. However, the number of mutant mice and the cell lines we generated were insufficient for genome-wide in-depth analyses. Furthermore, precise evaluation of the insertion preference was hampered by bias during mutagenesis such as the identification of gene hits by reporter gene expression.

Here, we report a large-scale genome-wide characterization of insertion site profiles of MLV, PB, Tol2 and SB. We carefully designed our experiments to minimize any selection bias during the detection of insertion sites. We utilized high-throughput DNA sequencing technologies to obtain a large number of insertion sites that are sufficient for statistical analyses. We used mouse ESCs as host cells because of the availability of a large dataset of genomic and epigenomic information such as gene expression, histone modifications, chromatin binding sites of various transcriptional regulators, and higher-order chromatin structures. Our analyses revealed that all vectors have a distinct insertion site preference. These results will be useful to determine the efficient utilization of each vector in functional genomics and gene therapy studies.

## Results

### Experimental design and consideration for unbiased analyses

**Figure 1:**
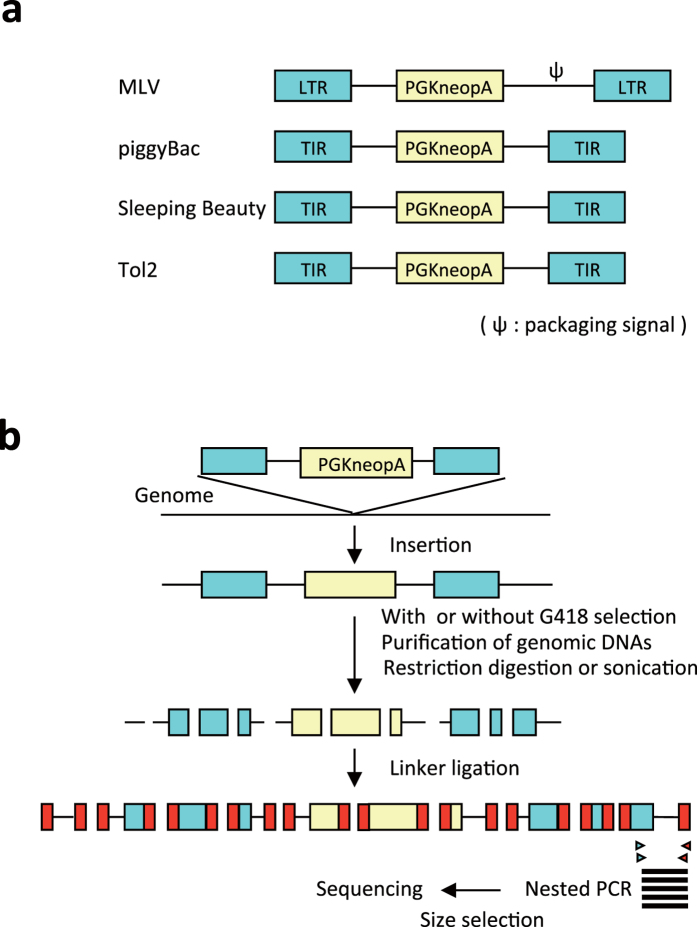
Strategy for the determination of vector insertion sites. (**a**) Vector structure. In the MLV vector, the PGKneopA cassette was placed in reverse orientation relative to viral transcription. TIR, terminal inverted repeat. (**b**) Procedure for the determination of vector insertion sites.

Figure 1 shows an overview of the analyses. The vector structures are shown in Fig. 1a and the experimental scheme to determine the insertion sites are presented in Fig. 1b. All vectors contained a neo gene expression cassette driven by a phosphoglycerate kinase-1 promoter^13^, which has widely been used in mouse ESCs.

To characterize insertion site profiles as precisely as possible, we listed the possible factors that may introduce bias during the analysis and considered solutions to this (Supplementary Table S1). First, we utilized high-throughput DNA sequencing methods to obtain a large dataset. We employed two complementary platforms, Roche GS FLX (Supplementary Table S2) and Illumina GA2 (Supplementary Table S3). We used both platforms in some experiments in order to assess whether any platform-specific bias existed. The dataset obtained with Roche GS FLX was used more often in the present study because its long read length allowed us to achieve accurate mapping on the mouse genome. Second, we tried to avoid the effect of transcriptional silencing of the neo selection marker. Neo selection markers can be silenced in some insertion sites such as heterochromatin regions, in which case such insertion sites would be lost during G418 selection and excluded from the downstream analysis. To avoid this bias, we set up a condition for multiple vector insertion per cell so that silencing of the neo gene in a given locus would be rescued by the expression of the neo gene in other loci. Southern blot analysis of G418-resistant ESC clones showed that the mean number of vector DNA per cell was 1.4, 4.4, 2.6, 3.0 in MLV, PB, Tol2, SB, respectively (Supplementary Fig. S1). We used Southern blot analysis to verify that vector DNA was not present in G418-sensitive clones (Supplementary Fig. S1), further confirming that the loss of vector-inserted clones was a rare event under our experimental conditions. As an alternative to avoid selection bias, we omitted G418 selection in some experiments and compared the results with and without selection. This evaluation was necessary, especially for MLV, because a high copy number of MLV insertions was hard to achieve in ESCs (Supplementary Fig. S1). Third, remobilization of integrated transposons can lead to the misinterpretation of insertion preference because DNA transposons hop locally as we and others reported previously^10^,^11^,^14^,^15^,^16^,^17^,^18^. In cases where transposons hopped locally after cell division, original insertion sites and re-insertion sites could be close to each other and misinterpreted as a hotspot for insertion. To avoid this, we conducted three independent transfections and assessed the insertion sites between different experiments. Fourth, PB and SB are inserted into TATA and TA sequences^7^,^8^, respectively, which inevitably constrains the distribution of insertion sites. Therefore, we also introduced this constraint in *in silico*-generated controls. Fifth, distribution of restriction sites used for genomic DNA fragmentation in Roche GS FLX may introduce bias. To avoid this, we reflected the distribution of the restriction site in *in silico* controls. Alternatively, we fragmented genomic DNAs with sonication in Illumina GA2 and compared the results with Roche GS FLX.

### Genome-wide distribution of vector insertion sites

**Figure 2:**
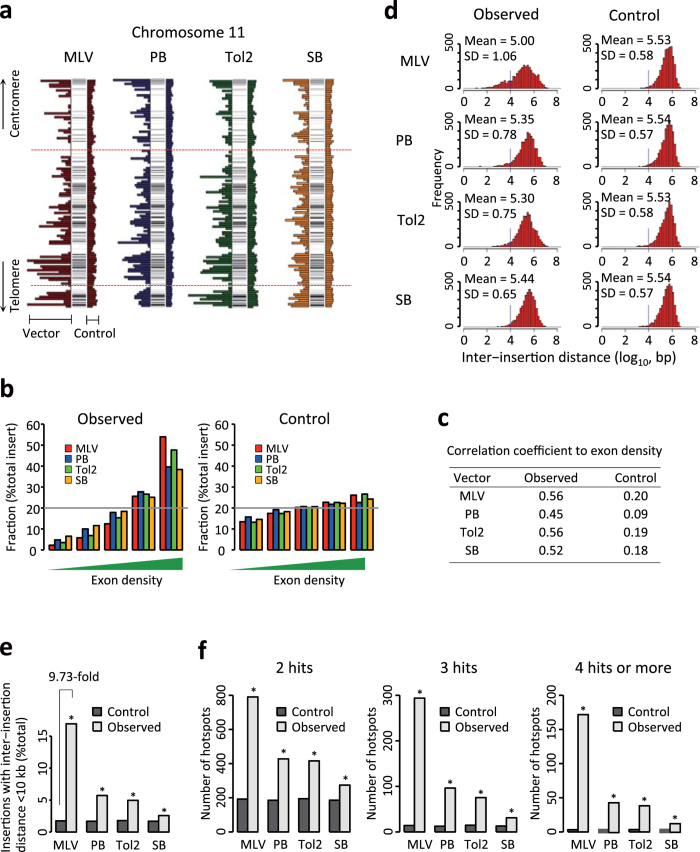
Chromosomal distribution of vector insertion sites and hotspot insertion sites. (**a**) Representative distribution of insertion sites on mouse chromosome 11. Horizontal stripes in the vertical bar indicate exon density. Insertion frequencies of MLV (red), PB (blue), Tol2 (green) and SB (yellow) are plotted on the left side of each vertical bar, and insertion frequencies of *in silico* control are shown on the right side. Red dotted lines indicate regions with poor insertion frequency for vectors and *in silico* controls. The data are presented in 1-Mbp windows. (**b**) Fraction of vector insertion by exon density. Genomic regions were divided into 500-kb bins and grouped into five ranks (lowest, low, mid, high, and highest) according to exon density. Insertion frequency was calculated from the observed data (left) and the *in silico* control data (right). (**c**) Pearson’s correlation coefficient (r) between exon density (total exon size in 500-kb window) and insertion density (number of inserts in 500-kb window). We compared the correlation coefficients of observed data to those of *in silico* control data using bootstrap method. All vectors showed significant increase of correlation coefficient (more inserts in exon rich regions) in observed data (*P* < 0.001). (**d**) Distribution of the distance between insertion sites. We sampled 4,000 insertions sites from each vector dataset 1,000 times by permutation and measured the distance between adjacent insertion sites. The X-axis shows bins of inter-insertion distance presented as log10 scale and the Y-axis indicates the number of cases in each bin. Mean and standard deviation (SD) are shown as log10 scale. Inter-insertion distances of 10-kb are indicated by a vertical line. Insertions to the left of this line are quantitated in (**e**). (**e**) Frequency of inter-insertion distances within 10-kb. (**f**) Hotspot insertion sites. Hotspots are defined as genomic regions fulfilling the following criteria: 2 hits within a 30-kb window (left), 3 hits within a 50-kb window (middle), or 4 or more hits within a 100-kb window (right). We sampled 4,000 insertion sites from each dataset 1,000 times and determined the mean number of hotspots as shown in the Y-axis. In (**e,f**), *P*-values were calculated by bootstrapping. *P < 0.001.

Following G418 selection of vector-inserted cells and restriction digestion of genomic DNAs (Fig. 1b), we determined insertion sites for MLV (n = 5,894,), PB (n = 6,368), Tol2 (n = 4,439), and SB (n = 8,641), using Roche GS FLX (Supplementary Table S2). Genome-wide distribution of the insertion sites was assessed in 1-Mb windows with exon distribution (Fig. 2a, Supplementary Fig. S2). Almost all genomic regions were inserted by all four vectors with this resolution. Of note, some genomic regions exhibited poor insertion events in all datasets including *in silico* controls (shown by red dotted lines in Fig. 2a). This observation indicates that comparison with the *in silico* control is essential for the precise evaluation of insertion site distribution. Although genome-wide insertion was observed by 1-Mb windows, insertion sites were not uniformly distributed and seemed biased toward exon rich regions (Fig. 2a, Supplementary Fig. S2). To validate the insertion preference toward exon-rich regions statistically, we divided the entire genome into 500-kb regions and categorized them into equal sized five groups by exon density: lowest, low, mid, high, and highest (Supplementary Fig. S3). Vector insertions were more frequently observed in regions with higher exon density compared to the *in silico* controls (Fig. 2b), demonstrating insertion bias toward exon-rich regions. We also calculated the correlation coefficients between the number of insertion sites and exon density for all vectors (Fig. 2c, range from 0.45 to 0.56). Matched control inserts showed markedly lower correlation coefficients (Fig. 2c, range from 0.09 to 0.20). To assess the insertion bias further, we analysed the distribution of the inter-insertion distances, distance between adjacent insertion sites (Fig. 2d,e). MLV showed significant enrichment of insertion sites at reduced inter-insertion distances compared to other vectors. (Fig. 2d,e, see Supplementary Table S4 for the inter-insertion distance and the statistical comparison between vectors). The number of less than 10-kb inter-insertion distances were 9.73-fold higher than in the matched control in MLV (Fig. 2e), suggesting MLV insertions are clustered within narrow genomic regions. We further assessed the distribution bias by quantifying hotspots of vector insertion (Fig. 2f, see Methods for the definition of hotspot). Consistent with the results of the inter-insertion distance (Fig. 2d,e), MLV showed the highest number of hotspots, followed by PB and Tol2, whereas SB showed the lowest number of hotspots (Fig. 2f, see Supplementary Table S5 for the number of hotspots and statistical comparison between vectors). Importantly, clusters of vector insertions in most hotspot regions derived from independent transfections (Supplementary Fig. S4). Therefore, we concluded that the local hopping feature of the transposons did not affect the determination of hotspot regions.

**Figure 3:**
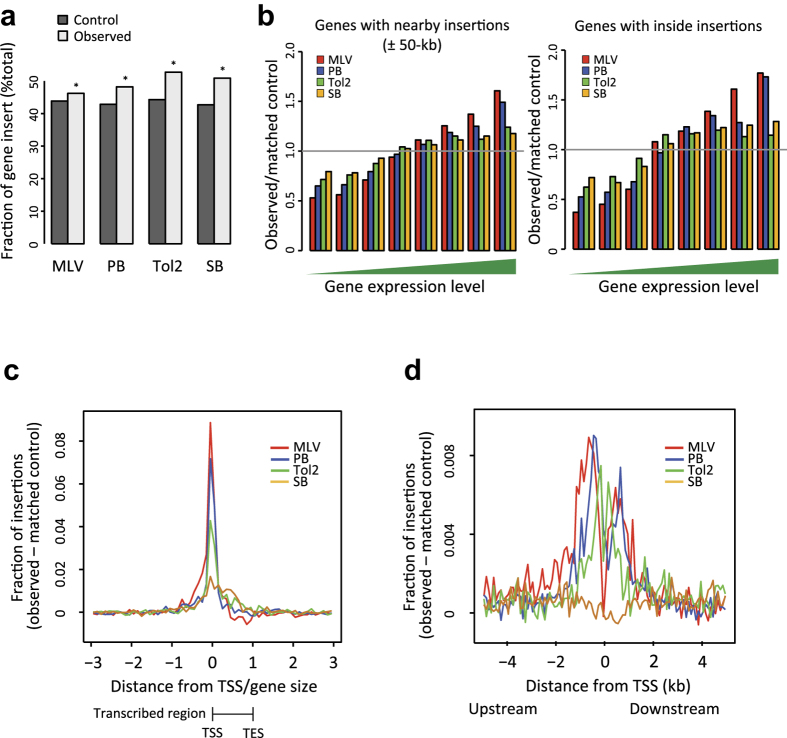
Relationship between insertion sites and RefSeq, gene expression, and transcription start sites. (**a**) Relative insertion frequency inside RefSeq genes. The Y-axis shows the ratio of the number of insertion sites inside RefSeq genes to that of the corresponding matched control. *P*-values were calculated by binomial statistics. *P < 0.001. (**b**) Insertion frequency relative to gene expression levels. The X-axis shows bins of increasing expression ranks in ESCs from lowest to highest. (**c**) Relative distance between TSS and vector insertion sites. The distance from TSS was divided by gene size and *in silico* control data was subtracted. TES, transcription end site. (**d**) Distribution of insertion sites ± 5-kb regions relative to TSS. *In silico* control data were subtracted from these values.

Because we observed insertion bias toward exon rich regions (Fig. 2a–c), we compared vector insertions by the features of genes near to insertion sites. First, we compared the insertion frequency inside RefSeq genes irrespective of their expression levels in ESCs. All vectors inserted inside genes with a similar frequency (Fig. 3a) including SB that showed minimum insertion bias in Fig. 2. However, substantial differences were observed when we classified genes by their expression levels in ESCs (Fig. 3b). MLV was highly biased to highly expressed genes, and PB showed a similar preference (Fig. 3b, see Supplementary Table S6 for statistical comparison between vectors). In contrast, gene expressions were weaker at Tol2 and SB insertion sites (Fig. 3b, Supplementary Table S6). The difference between PB and Tol2 was unexpected because the frequency of hotspots was similar between them (Fig. 2f, Supplementary Table S5). We also analysed the distribution of insertion sites relative to the transcribed region of the RefSeq genes (Fig. 3c). MLV, PB and Tol2 showed a preference for TSS, whereas SB insertion did not correlate with TSS and was slightly enriched throughout transcribed regions (Fig. 3c). We therefore conducted high-resolution analysis around TSS and revealed the bimodal distribution of MLV, PB, Tol2 insertions: the peak of the distribution was not exactly at the TSS but rather upstream and downstream of TSS (Fig. 3d). Notably, the insertion frequency of MLV at TSS was lower than for PB and Tol2 and was close to the basal level (Fig. 3d). These results indicate that the vector insertion profile was influenced by factors other than gene expression levels. These findings were not biased by G418 selection because similar results were obtained without G418 selection (Supplementary Table S2, Supplementary Fig. S5). We also confirmed that the Illumina GA2 analyses (*Wt* in Supplementary Table S3) gave similar results to the Roche GS FLX (Supplementary Fig. S6). Because genomic DNAs were fragmented by sonication in Illumina GA2 instead of restriction digestion, this also indicates that DNA fragmentation methods did not introduce bias to the analyses of vector insertion preference.

The results in Fig. 3 indicated that each vector was influenced by different factors such as gene expression levels and TSS, suggesting that the insertion of each vector is regulated by different mechanisms.

### Distribution of vector insertions at histone modifications

**Figure 4:**
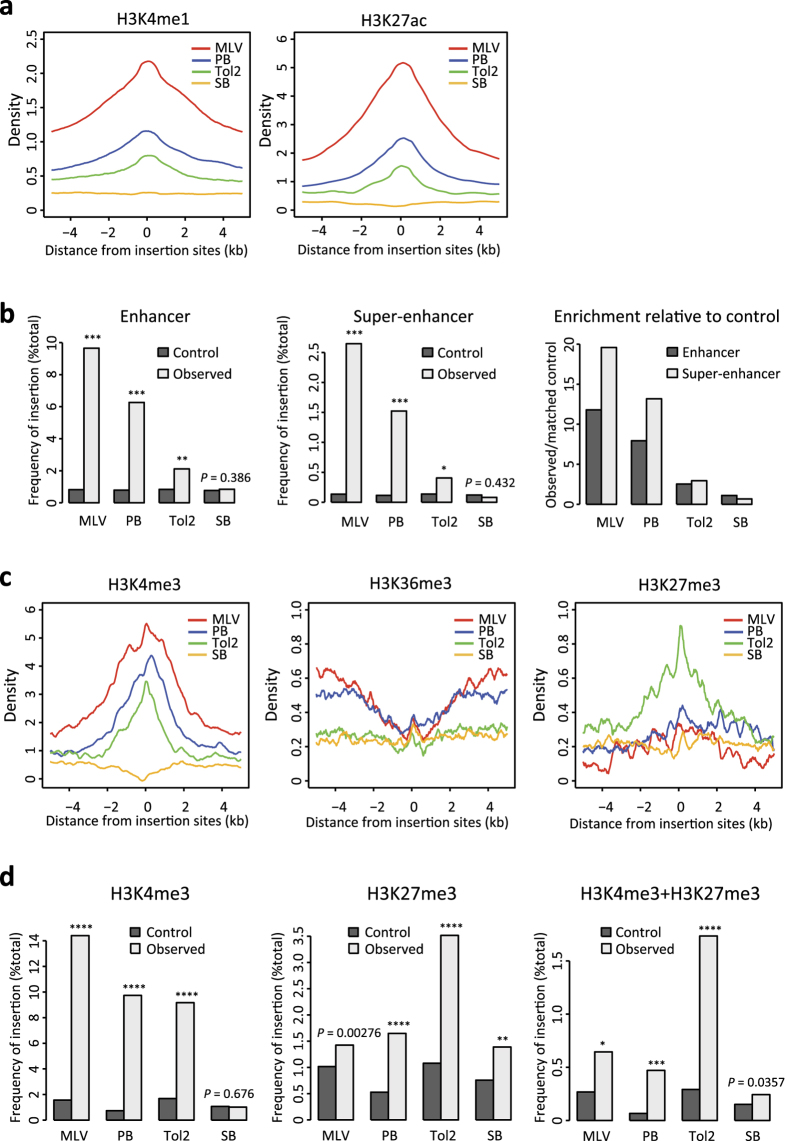
Distinct overlapping patterns between vector insertion sites and histone modification sites. (**a**) Line plots of enhancer type histone modification in ± 5-kb regions relative to insertion sites. The panels show the mean density values after the subtraction of control values. (**b**) Enrichment of vector insertion sites at enhancers or super-enhancers. The number of insertion sites located inside enhancer or super-enhancer regions were compared to the expected number of insertion sites obtained from matched controls. *P*-values were calculated by binomial test and adjusted by FDR for multiple comparisons. *P < 10^−4^, **P < 10^−14^, ***P < 10^−71^. (**c**) Line plots of histone modifications representing transcriptional status in ± 5-kb regions relative to insertion sites. (**d**) Bar plots of histone marks relative to insertion sites. We used public datasets of histone modification-enriched regions to calculate the fraction of insertion sites located in each region as shown in the Y-axis (see Methods for details). *P*-values were calculated by binomial test and adjusted by FDR for multiple comparisons. *P < 10^−5^, **P < 10^−8^, ***P < 10^−15^, ****P < 10^−21^.

Recent studies demonstrated that gene expression profiles are largely determined by histone modifications^19^. Whereas mRNA levels reflect the current state of gene expression, histone modification patterns provide further information such as the inducibility of genes upon stimulation or the underlying mechanisms of gene activities. Therefore, we considered that the analysis of histone modification might provide mechanistic insights into the correlation of vector insertions with gene expression profiles. We analysed the enhancer markers H3K4me1 and H3K27ac (Fig. 4a) and found that MLV insertion was highly enriched in the regions with both markers, followed by PB and Tol2. In contrast, SB showed no significant enrichment in regions with either marker. Recent studies revealed a new class of enhancer called a “super-enhancer”, which is a large cluster of enhancers characterized by an unusually high level of Mediator binding^20^. MLV and PB insertions were significantly enriched in both conventional enhancers and super-enhancers compared to *in silico*-generated matched controls (Fig. 4b, left and middle, see Supplementary Table S7 for statistical comparison between vectors), and the degree of enrichment in super-enhancers was greater than that in conventional enhancers (Fig. 4b, right). In contrast, the degree of enrichment of Tol2 and SB insertions in super-enhancers was similar to that in conventional enhancers (Fig. 4b, right). These results indicate a preference of MLV and PB for strong enhancers.

Next, we investigated the relationship of vector insertion with histone modifications that are highly associated with transcriptional status (H3K4me3, H3K36me3, and H3K27me3) (Fig. 4c,d). H3K4me3 is enriched at active TSS, H3K36me3 accumulates in transcribed regions, and H3K27me3 is a repressive mark introduced by the Polycomb repressive complex^21^. Substantial enrichment of H3K4me3 was observed for MLV and PB (Fig. 4c,d, see Supplementary Table S8 for statistical comparison between vectors). This result is consistent with the preference of MLV and PB for TSS regions (Fig. 3c,d). Accumulation of H3K36me3 was observed at the distal region of MLV and PB insertion sites (Fig. 4c). This can be explained by the insertion preference of MLV and PB near transcriptionally active TSSs. In contrast, H3K4me3 deposition was not observed at SB insertion sites (Fig. 4c,d). This result is consistent with the observation that SB insertion is not skewed to TSS (Fig. 3c,d). Similar results were obtained with and without G418 selection (Supplementary Fig. S5), indicating G418 selection did not introduce bias. H3K36me3 was not enriched at SB insertion sites (Fig. 4c), consistent with the weak relationship of SB insertion with gene expression (Fig. 3b). By contrast, Tol2 insertion sites exhibited a unique association pattern with histone modifications. Although H3K4me3 was accumulated at Tol2 insertion sites, no apparent accumulation was observed for H3K36me3 (Fig. 4c). This suggests that TSSs around Tol2 insertion are active for transcription initiation but inactive for transcription elongation. The most intriguing observation was the enrichment of Tol2 insertion sites at H3K27me3-modified regions (Fig. 4c,d). It has been reported that substantial fractions of genes are marked by both H3K4me3 and H3K27me3 in ESCs^19^,^21^. This bivalent modification is considered to represent a poised state of gene expression, which indicates that these genes are silent in undifferentiated ESCs and induced upon differentiation^19^,^21^. To investigate whether Tol2 insertion sites are enriched for bivalent modification of H3K4me3 and H3K27me3, we used a previously reported dataset of the bivalent regions^19^. Tol2 insertion was most highly enriched in bivalent regions among the four vectors (Fig. 4d, Supplementary Table S8), strongly suggesting that Tol2 has a preference for the transcriptionally poised chromatin state.

### Insertion preference at developmentally regulated genes

**Figure 5:**
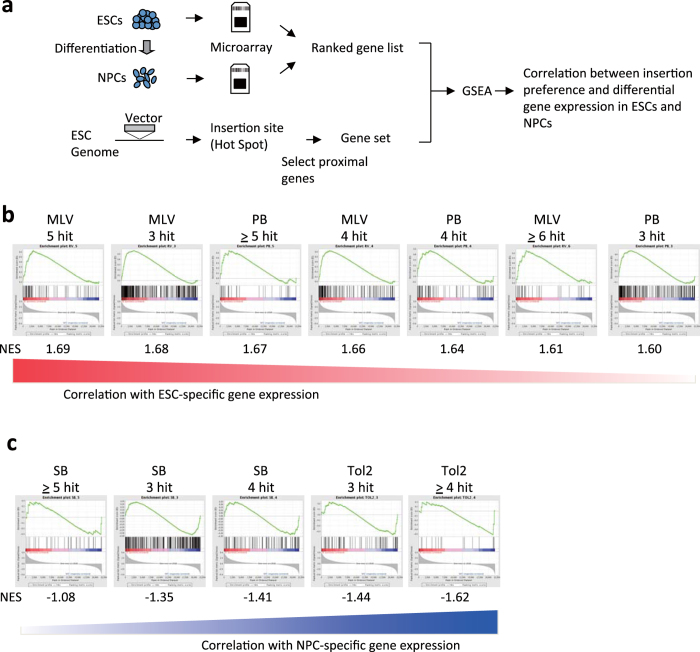
Distinct insertion preference in developmentally regulated genes. (**a**) Overview of Gene Set Enrichment Analysis (GSEA). (**b**, **c**) Correlation of gene sets with ESC-specific gene expression (**b**) and NPC-specific gene expression (**c**). NES, normalized enrichment score.

Distinct distribution patterns of the four vectors relative to the status of histone modifications (Fig. 4) suggest that the target genes of each vector might exhibit different expression profiles during development. To address this, we conducted Gene Set Enrichment Analysis (GSEA)^22^ using a microarray dataset of ESCs and NPCs (Fig. 5a). Genes at the insertion hotspots were grouped as a gene set and rank-ordered by expression specificity in ESCs and NPCs (Fig. 5a). Gene sets frequently observed in MLV and PB insertion sites were enriched with ESC-specific genes (Fig. 5b). This result is consistent with the observation that both MLV and PB insertions were enriched in ESC-super-enhancer regions (Fig. 4b) that determine the cell identity of ESCs^20^,^23^. In contrast, gene sets frequently observed in Tol2 and SB insertion sites were enriched with NPC-specific genes (Fig. 5c), indicating that the insertion sites of Tol2 and SB are enriched with genes that are silent or weakly expressed in undifferentiated ESCs and induced upon differentiation into NPCs. Notably, this association was most strongly observed for Tol2. This result is consistent with the observation that Tol2 insertion sites are enriched with bivalent histone modification (Fig. 4d), which is a marker of the transcriptionally poised state and is often observed in inducible genes^21^. These results demonstrate that each vector prefers a distinct class of developmentally regulated genes.

### Distinct distribution of insertion sites relative to open chromatin

**Figure 6:**
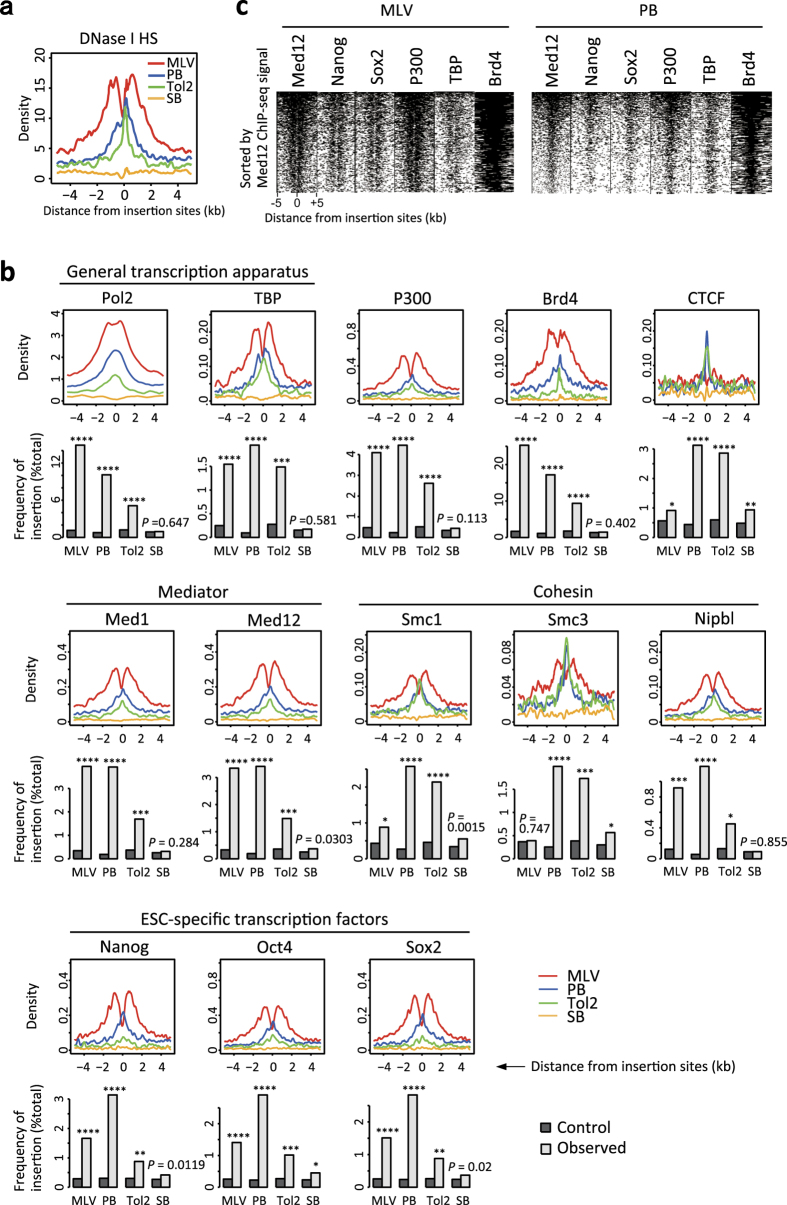
Differential distribution of insertion sites relative to transcriptional regulatory proteins. (**a**,**b**) Line plots of DNase I HSs (**a**) and the density of transcriptional regulatory proteins (**b**) in ± 5-kb regions relative to vector insertion sites. The panels show the means of density values after subtraction of control values. *P*-values were calculated by binomial test and adjusted by FDR for multiple comparisons. *P < 10^−3^, **P < 10^−6^, ***P < 10^−10^, ****P < 10^−30^. (**c**) Density map of regulatory proteins in ± 5-kb regions relative to the insertion sites of MLV (left) and PB (right). Each row represents one insertion site, and the insertion sites are located at the centre of these plots. We determined whether the insertion sites overlap with the regulatory protein binding sites. Areas overlapping or not with the binding sites, are shown in black or white, respectively. The insertion sites are sorted by the total width of peak regions of Med12 within ± 5-kb from the insertions site.

Vector insertion profiles were highly correlated with gene expression levels (Figs 3b and 5). Because expressed genes are considered to be present in open chromatin regions, we investigated the correlation between insertion sites and DNase I hypersensitive sites (DNase I HSs). Surprisingly, distributions of DNase I HSs were different for all vectors (Fig. 6a). Although both MLV and PB preferred highly expressed genes (Figs 3b and 5), the distribution of DNase I HSs was bimodal in MLV, whereas a single peak was seen at the PB insertion site (Fig. 6a). Both Tol2 and SB showed weak association with ESC-expressed genes (Figs 3b and 5). However, a sharp narrow peak of DNase I HSs was observed at the Tol2 insertion site, whereas almost no correlation was seen in SB (Fig. 6a). These results indicate that open chromatin is not the sole determinant of vector insertion sites.

### Distinct distribution of insertion sites relative to transcriptional regulators involved in 3D chromatin structures

Because open chromatin was only partially associated with insertion site preference, we investigated the relationship of insertion sites with 3D chromatin structures. Recent studies revealed the hierarchy of chromatin structure and its effect on gene expression^24^,^25^,^26^,^27^. Large megabase-sized chromatin loops, termed topologically associating domains (TADs), are formed by CTCF and Cohesin. Within TADs, there are smaller loops formed by Cohesin and Mediator, which contribute to enhancer–promoter interactions. Within enhancer–promoter loops, general transcription apparatus or cell-type specific transcription factors are clustered. Therefore, we analysed the relationship of insertion sites and DNA-binding sites of these regulatory proteins. The following proteins were analysed: general transcription apparatus (Pol2, TBP), Mediator (Med1, Med12), Cohesin (Smc1, Smc3, Nipbl), insulator protein (CTCF), enhancer binding protein (P300), ESC-specific transcription factors (Nanog, Oct4, Sox2) and Brd4. Brd4 is a member of the BET (bromodomain and extra-terminal domain) family. Brd2, Brd3 and Brd4 were recently reported to interact with retrovirus integrase and to recruit the retroviral preintegration complex to the insertion site^28^,^29^.

These analyses revealed a relationship of vector insertion with various factors involved in 3D chromatin structures (Fig. 6b, see Supplementary Table S9 for statistical comparison between vectors). PB insertion was highly enriched at the DNA-binding sites of Cohesin, Mediator, and CTCF, which play critical roles in the formation of anchor sites of TADs (Fig. 6b). PB was also highly enriched at the DNA-binding sites of general transcription apparatus and ESC-specific transcription factors (Fig. 6b), suggesting that PB-inserted chromatin loops are transcriptionally active. MLV insertion was also associated with general transcription apparatus, ESC-specific transcription factors, Cohesion, and Mediator (Fig. 6b). However, a marked difference was observed in CTCF: There was a sharp peak of CTCF-enrichment at the PB insertion site, whereas such a peak was not observed in MLV (Fig. 6b), suggesting MLV prefers smaller-size chromatin loops representing enhancer-promoter interactions^24^,^25^,^26^,^27^. Tol2 insertions were highly enriched at Cohesin (Smc1 and Smc3) and CTCF as in the case of PB. There was no difference between the enrichment at CTCF, Smc1, and Smc3 binding sites between PB and Tol2 (Fig. 6b, Supplementary Table S9, *P*-values were Smc1 = 0.160, Smc3 = 0.351, and CTCF = 0.549 by Fisher’s exact test). The result indicates insertion preference for the anchor sites of chromatin loops in PB and Tol2. In contrast, correlations of Tol2 with Mediator and ESC-specific transcription factors were weaker than those of PB (Fig. 6b, Supplementary Table S9, *P*-values comparing the difference in enrichment between Tol2 and PB were <10^−9^ for Med1, Med12, Nanog, Oct4, and Sox2 binding sites by Fisher’s exact test), suggesting that Tol2-inserted chromatin loops harbour transcriptionally poised or weakly expressed genes. This idea is consistent with the preference of Tol2 to bivalent histone modifications that are present at silent and inducible loci (Fig. 4d). Of note, SB showed almost no or only poor correlation with all chromatin states examined (Fig. 6b, Supplementary Table S9).

Interestingly, chromatin occupancy of regulatory proteins was highest at the insertion sites of PB and Tol2. In contrast, MLV showed a bimodal distribution pattern (Fig. 6b) as in DNase I HSs (Fig. 6a). This bimodal distribution was also observed in Brd4 (Fig. 6b), which is reported to be an interactor of retroviral integrase^28^,^29^. These results suggest that MLV is inserted at a distant location after being recruited by Brd4.

One of the intriguing observations in Fig. 6b is that most transcriptional regulatory proteins showed similar distribution patterns. This suggests that the chromatin binding sites of these proteins may form a cluster. To investigate this, we aligned ChIP-seq signals of the regulatory proteins side-by-side around 5-kb regions of each insertion site (Fig. 6c). All regulatory proteins showed a similar distribution pattern: bimodal distribution was observed in MLV, whereas the signals were strongest near the insertion sites in PB, indicating that the DNA-binding sites of the regulatory proteins were indeed clustered.

### Perturbation of chromatin states and its effect on vector insertion profiles

**Figure 7:**
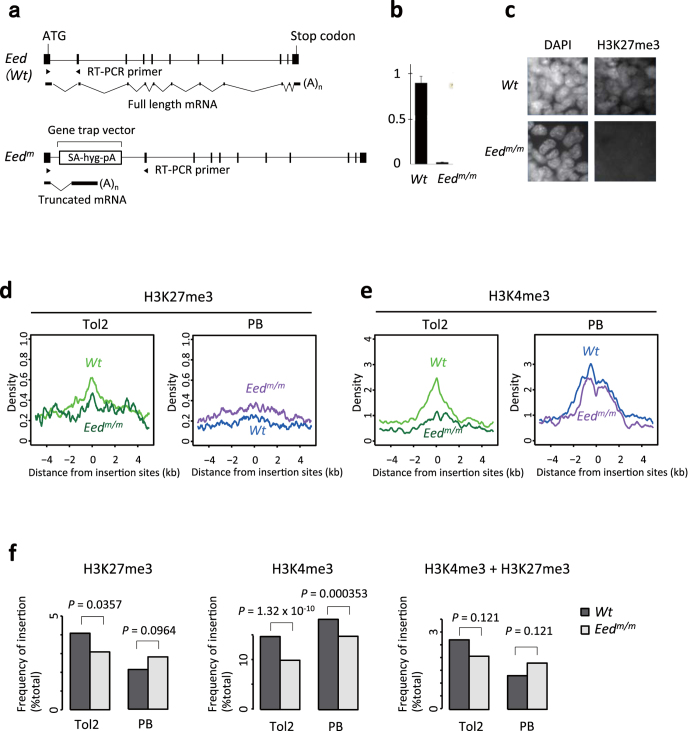
Alteration of the preference of vector insertion in *Eed*-homozygous mutant ESCs. (**a**) Disruption of *Eed* by gene trap. *Eed*^*m*^ indicates mutant allele. For the gene trap vector, only relevant elements for gene disruption are presented. *Wt*, wild-type; *m*, mutation; SA, splice acceptor; hyg, hygromycin-resistance gene; pA, polyadenylation signal. (**b**) qRT-PCR of *Eed* expression for wild-type ESCs (*Wt*) and homozygous mutant ESCs (*Eed*^*m/m*^). Locations of the PCR primers are shown in (**a**). Error bars indicate standard deviation. (**c**) Immunostaining of H3K27me3. (**d,e**) Line plots of histone modifications in ± 5-kb regions relative to insertion sites. The panels show the means of density values after the subtraction of control values. (**f**) Bar plots of histone marks relative to insertion sites. We compared the number of insertion sites located within ± 2-kb from histone modification-enriched regions between *Wt* and *Eed*^*m/m*^ ESCs by Fisher’s exact test and adjusted by FDR for multiple comparisons. In (**d-f**), public datasets of histone modification-enriched regions of *Wt* ESCs were used as in the case of Fig. 4c and d. *P*-values were calculated by Fisher’s exact test and adjusted by FDR for multiple comparisons.

The results described above suggest that chromatin states are major determinants of vector insertion profiles. To validate this idea, we perturbed the chromatin states of ESCs and investigated its effect on vector insertion distribution. Among various chromatin states analysed in this study, we focused on H3K27me3 because the insertion preference of Tol2 in H3K27me3-modified regions was one of the most notable and unexpected findings (Fig. 4c,d). The trimethylation of H3K27 is catalysed by Polycomb repressive complex 2 including Eed as an essential component^30^. We therefore introduced Tol2 or PB transposons into a homozygous *Eed*-mutant (*Eed*^*m/m*^) ESC line, which is present in our previously reported mutant ESC bank^5^. After confirming the loss of H3K27me3 modification in *Eed*^*m/m*^ ESCs (Fig. 7a–c), we determined the distribution patterns of Tol2 and PB in *Eed*^*m/m*^ ESCs using the Illumina platform (Supplementary Table S3) and compared them with those in wild-type (*Wt*) ESCs (Fig. 7d–f) with regards to chromatin states. As a reference for the genome-wide distribution pattern of histone modifications (H3K27me3 and H3K4me3), we used public ChIP-seq datasets from *Wt* ESCs^19^. As expected, homozygous knockout of Eed decreased the enrichment of Tol2 insertions in the regions corresponding to the H3K27me3 marks in the *Wt* genome (Fig. 7d,f, left, *P* = 0.0357 by Fisher’s exact test), which is consistent with the idea that the H3K27me3 mark is an important determinant of Tol2 insertion. In contrast, the frequency of PB insertion at H3K27me3-modified regions did not decrease in *Eed*^*m/m*^ ESCs (Fig. 7d,f, left), supporting the specific effect of H3K27me3 on Tol2 insertion. Of note, this insertion site preference was not completely abolished in *Eed*^*m/m*^ ESCs (Fig. 7d), indicating that H3K27me3 itself is not the direct target of Tol2 insertion. Other epigenetic modifications associated with H3K27me3 could remain and enhance Tol2 insertion in their corresponding regions even after the disruption of Eed. Unexpectedly, we observed significant reduction of Tol2 and PB insertions in the H3K4me3-modified regions in *Eed*^*m/m*^ ESCs (Fig. 7e,f, middle), which was more evident in Tol2 than in PB. One possible explanation for this observation is that the homozygous knockout of Eed disrupted the H3K4me3 and H3K27me3 bivalent modification, which is more strongly associated with Tol2 insertion rather than with PB insertion (Fig. 4c,d). However, no significant reduction of Tol2 and PB enrichment was observed in the bivalent regions in *Eed*^*m/m*^ ESCs (Fig. 7f, right). It has been reported that the elimination of H3K27me3-modification by Eed-knockout alters the expression levels of a large number of developmental regulators in mouse ESCs^30^. We therefore hypothesize that secondary or additional changes in chromatin states associated with the loss of H3K27me3 could also affect the insertion preferences of Tol2 and PB in the H3K4me3-modified regions.

**Figure 8:**
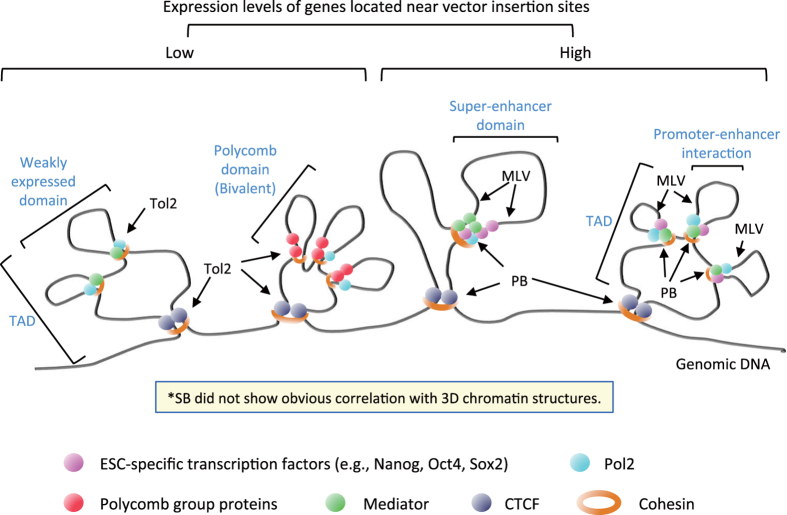
Schematic model of the distinct insertion profiles of MLV, PB, Tol2 and SB. Insertion preference for each vector is illustrated in terms of the hierarchical 3D structure of chromatin. Note that SB showed almost no insertion preference.

These data from *Eed*^*m/m*^ ESCs suggest that chromatin state affects vector insertion profiles. Based on the various chromatin states investigated in the present study, we summarized the characteristic features of each vector insertion in Fig. 8 (see Discussion for details). We also summarized the observations in the present study and previous reports in Supplementary Table S10 to clarify our novel findings.

## Discussion

Vector insertion preference has been investigated extensively in previous studies (Supplementary Table 10) 17,31,32,33,34,35,36,37,38,39,40,41,42,43,44. The main difference between our study and previous studies is that we analysed four vectors systematically under the same experimental conditions. Furthermore, we interpreted our results based on various factors involved in the 3D organization of chromatin (Fig. 8).

We observed a distinct association between vector insertion preference and chromatin structural proteins, e.g., Cohesin and CTCF^24^,^25^,^26^,^27^ (Fig. 8). Cohesin is a ring form protein complex and cooperates with a DNA-binding protein CTCF to make a megabase-sized DNA loop called a topologically associating domain, TAD. There are smaller DNA loops within TADs that represents the promoter–enhancer interaction of embedded genes. Cohesin forms this smaller-size promoter–enhancer interacting loop without CTCF^24^,^25^,^26^,^27^. Both PB and MLV insertion sites showed a correlation with Cohesin (Fig. 6b). Considering the significant insertion preference of PB and MLV into TSS and enhancer regions (Figs 3c,d and 4a,b), it is highly likely that the correlation with Cohesin represents an insertion preference for the anchor areas of the promoter–enhancer loops (Fig. 8). However, correlation of PB with CTCF was much higher than MLV (Fig. 6b, Supplementary Table S9, *P*-value between PB and MLV = 2.41 × 10^−18^ by Fisher’s exact test), suggesting PB has a preference for the anchor areas of both promoter–enhancer loops and TADs, whereas MLV prefers promoter–enhancer loops (Fig. 8). This observation is consistent with the result that MLV had more hotspots than other vectors (Fig. 2f). A recent report indicated similarities between insertion site distributions of PB and MLV^34^. Our result is consistent with their report and further revealed a substantial difference between PB and MLV in the light of 3D chromatin structure.

Tol2 insertion correlated with both Cohesin and CTCF to a similar high level as observed for PB (Fig. 6b), suggesting that Tol2 prefers anchor areas of both promoter–enhancer loops and TADs (Fig. 8). However, Tol2 showed a weak correlation with Mediator and ESC-specific transcription factors (Fig. 6b). This result strongly suggests that Tol2-inserted loops are transcriptionally weak (Fig. 8). Transcriptionally active and inactive genes are often clustered in the genome, and TADs delineate the boundaries of the clusters^25^. It was reported that transcriptionally silent TADs are enriched by Polycomb repressive complexes^25^. Consistent with this idea, Tol2 insertion was associated with the bivalent modification of H3K4me3 and H3K27me3 (Fig. 4d). We speculate that PB and MLV prefer transcriptionally active chromatin loops, whereas Tol2 prefers transcriptionally weak chromatin loops (Fig. 8).

The results of Tol2 insertion preference need to be interpreted carefully. First, the preference of Tol2 for H3K27me3 in ESCs does not indicate insertion into heterochromatin regions. We speculate that the Tol2 preference for H3K27me3 is caused by coexisting modification with H3K4me3 in ESCs. Indeed, Tol2 insertion was reported to be inversely correlated with H3K27me3 modification in HeLa cells^31^. H3K27me3-modified regions are probably heterochromatinised in HeLa cells and antagonize Tol2 insertion. Second, Tol2 insertion is not restricted to bivalent regions because Tol2 also inserted into expressed genes although the expression levels were weaker than for PB and MLV (Fig. 3b).

Interestingly, most transcriptional regulators showed similar correlation patterns (Fig. 6b). This result is consistent with previous reports that a large protein complex of transcriptional regulators is present at the anchor site of TADs and promoter–enhancer loops^24^,^25^,^26^,^27^. These transcriptional regulators showed a binding peak at the insertion site of PB and Tol2, whereas the binding peak relative to the MLV insertion site was bimodal and several hundred bases away from the insertion site (Fig. 6b). Recent reports have demonstrated that Brd proteins interact with MLV integrase and define the insertion site^28^,^29^. However, our results indicated that the Brd4 binding peak was also bimodal similar to other transcriptional regulators and did not coincide with the MLV insertion site (Fig. 6b). We speculate that MLV integrase cannot replace the large protein complex at the anchor region and insert into adjacent less crowded regions. A recent report demonstrated that the PB transposase also interacts with Brd proteins^34^. It would be interesting to investigate whether the Tol2 transposase also interacts with Brd proteins because both PB and Tol2 correlated with Brd4 in our analysis (Fig. 6b).

To validate the role of chromatin states on vector insertion profiles, we investigated the distribution of vector insertion sites in *Eed*-homozygous mutant ESCs and demonstrated that vector insertion profiles changed in the absence of H3K27me3 mark (Fig. 7). To further assess the effect of chromatin states on vector insertion profiles, it would be interesting to induce redistribution of chromatin marks (not the loss of chromatin marks as in the case of *Eed*-homozygous mutant ESCs) and examine its effect on vector insertions. ESCs acquire a naive ground state when cultured in 2i media (serum-free medium with Gsk3/Mek inhibitors)^45^ and undergo substantial chromatin remodeling^46^,^47^,^48^. Chromatin states will also be altered when ESCs are differentiated into various cell lineages. Analysis of vector insertions in these culture conditions will provide further insights into the relationship between chromatin states and vector insertion.

Interestingly, SB insertion showed almost no relationship with any genomic and epigenomic features (Figs 2, 3, 4, 5, 6 and 8). Furthermore, SB insertion showed no correlation with DNase I HSs (Fig. 6a). This result suggests that SB can access tightly packed chromatin regions. This idea is consistent with our previous observation that SB transposase can efficiently excise the SB transposon from heterochromatin regions^49^,^50^. A previous study demonstrated that the SB insertion site was affected by sequence-dependent local DNA conformation^32^. This report together with our current results indicates the unique characteristics of SB when interacting with the host genome. It should be noted that SB transposase has been improved by extensive mutagenesis^51^. We utilized HSB^52^ in the present analyses. We speculate that other versions of SB transposases will also, if not all, have similar properties because a recent report demonstrated minimum insertion bias of the highly active SB100X transposase^51^ in human CD4^+^ T cells compared to MLV, PB and HIV^34^.

The current results provide valuable information for the application of each vector in various experimental settings. All vectors have been used as a mutagen for insertional mutagenesis^4^,^5^,^11^,^12^. The current results indicate that each vector targets distinct sets of genes suggesting that the combination of different vectors will expand the number of target genes. PB and MLV effectively target highly expressed genes, whereas Tol2 and SB would be appropriate for targeting moderately expressed or silent genes. This idea is supported by our previous experience in the construction of a mouse mutant ESC library in which the coverage of the mutation was expanded by the combination of retrovirus and Tol2^5^. A neutral feature of SB insertion will be essential if unbiased vector insertion is required. We previously utilized an enhancer trap-type SB vector for the saturated tagging of cis-regulatory elements of the *Pax1* gene in mouse ESCs^53^. Although expression of the *Pax1* gene is extremely low in ESCs, cis-regulatory elements were successfully tagged by the SB vector, indicating the advantage of the neutral feature of SB insertion.

Retroviral vectors are the most widely used vector for gene therapy when long-term gene expression is needed. Non-viral vectors are emerging as a promising alternative because of the simplicity of clinical grade sample preparation and their cost-effectiveness compared with viral vectors^1^. Indeed, all three transposon vectors analysed in the present study have been used successfully for the introduction of a chimeric antigen receptor into human T cells to redirect their antigen specificity toward cancer cells^1^,^54^,^55^. One of the major factors for the selection of gene therapy vectors is their insertion site preference. A recent report of clonal expansion of HIV-infected lymphocytes in human patients suggested that the adverse effect of vector insertion needs to be carefully monitored in gene therapy^56^. The safety issue of vector insertion has been addressed mainly by the insertion site proximity to transcriptional units^1^. The relative location of the vector insertion site to regulatory regions analysed in this study will help further assess safety during vector selection.

In summary, the distinct features of vector insertion profiles revealed in this study provide valuable information for the application of transposon and retroviral vectors for use in genetic engineering and gene therapy.

## Methods

### Vector construction

To construct the retroviral vector pCMT-PGKneo, a *Pac*I-*Xho*I fragment containing the floxed PGK-neo-pA cassette was excised from pMulti-ND-1.0^57^ and cloned into the *Pac*I-*Xho*I sites of pCMT-SAhygpA^5^.

The PB transposon vector pPB-PGKneo was constructed as follows. First, the 3′ terminal repeat of the PB transposon was amplified by PCR from pPB-SB-SA-bgeo^58^ using primers PB-P1 (CGACTCACTATAGGGCGAATTGGAGCTAGG) and PB-P5 (GCCGATATCAGATCTCTCGAGGAATTCGTTTAAACGGGCCCTTTGTTACTTTATAGAAGAAATTTTGAG). The PCR product was digested with *Asc*I and *Eco*RV and ligated to the *Asc*I-*Eco*RV fragment of pPB-SB-SA-bgeo containing the 5′ terminal repeat of the PB transposon and the plasmid backbone, resulting in PB-MCS-P5. Next, the *Pme*I–*Xho*I fragment of the pMulti-ND-1.0 containing the floxed PGK-neo-pA cassette was inserted into the *Pme*I-*Xho*I sites of the pPB-MCS-P5, resulting in pPB-PGKneo.

To construct the Tol2 transposon vector pTL2-PGKneo, the *Pac*I (blunt)-*Xho*I fragment containing the floxed PGK-neo-pA cassette was released from pMulti-ND-1.0 and cloned into the *Bgl*II (blunt)-*Xho*I sites of pT2AL200R150G^59^.

To construct the SB transposon vector pSB-PT2-PGKneo, the *Pme*I-*Not*I fragment containing the floxed PGK-neo-pA cassette was excised from pMulti-ND-1.0 and cloned into the *Eco*RV-*Not*I sites of pT2/HB^14^.

To construct the PB transposase expression vector pCAGGS-mPB, an *Eco*RI linker was inserted into the unique *Xho*I site of mPB^58^. The PB transposase-containing fragment was then released by *Eco*RI digestion and cloned into the *Eco*RI site of pCAGGS-EGFP^60^, resulting in pCAGGS-mPB.

To construct the SB transposase expression vector pCAGGS-HSB2, an *Eco*RI linker was inserted into the unique *Bam*HI site of pCMV-HSB2^52^. The SB transposase-containing fragment was then released by *Eco*RI digestion and cloned into the *Eco*RI site of pCAGGS-EGFP, resulting in pCAGGS-HSB2.

### Cell culture, transfection of transposons and infection of retrovirus

The V6.5 F1 hybrid mouse ESC line (C57BL/6 × 129S4/SvJae)^61^ was cultured in Knockout Dulbecco’s modified Eagle’s medium (Thermo Fisher Scientific, Waltham, MA, USA) supplemented with 20% foetal bovine serum, 0.1 mM nonessential amino acids, 0.1 mM 2-mercaptoethanol, 100 U/ml penicillin, 100 μg/ml streptomycin, 292 μg/ml L-glutamine and 1,000 U/ml leukemia inhibitory factor (EMD Millipore, Darmstadt, Germany) on mitomycin C-treated mouse embryonic fibroblast (MEF) feeder cells. *Eed*-homozygous mutant ESCs were generated from vdR2-4, a derivative of V6.5, and the details for the method of derivation was described previously^5^.

We conducted three independent transfections for each transposon. On day 0, 2 × 10^6^ ESCs were transfected with 10 μg of the transposon vector and 10 μg of the transposase expression vector using 120 μl of TransFast reagent (Promega, Madison, WI, USA), and plated onto one 6-cm dish. The following combinations of transposon and transposase vectors were utilized: pPB-PGKneo and pCAGGS-mPB, pTL2-PGKneo and pCAGGS-T2TP^9^, and pSB-PT2-PGKneo and pCAGGS-HSB2. In case ESCs were selected by G418, each cell population was passaged to two 10-cm dishes on day 2 and cultured in the presence or absence of G418 (150 μg/ml). ESCs were passaged at a ratio of 1:2 when they reached confluence. On day 9, MEFs were removed by plating ESCs on a gelatin-coated dish for 30 min and unattached cells were transferred onto a fresh dish. On day 11, ESCs were lysed and genomic DNAs were extracted. In case ESCs were not selected by G418, MEFs were removed on day 2 and ESCs were lysed on day 3. To infect ESCs with the MLV vector, Plat-E packaging cells^62^ were transfected with the MLV vector using Lipofectamine 2000 (Thermo Fisher Scientific). Two days later, viral supernatants were filtered through a 0.45-μm pore-size membrane and used to infect ESCs. The culture protocol following infection was same as that used for the transfection of transposon vectors.

### Quantitative RT-PCR

Total RNAs were extracted with TRIzol (Thermo Fisher Scientific) and treated with TURBO DNA-free kit (Thermo Fisher Scientific) to remove contaminating genomic DNAs. Eight hundreds ng of total RNAs were reverse transcribed with SuperScript IV (Thermo Fisher Scientific) using random primers (Promega). Expression levels of mRNAs encoding *Eed* and *β-actin* were quantified by real-time PCR using LightCycler FastStart DNA Master SYBR Green I kit (Roche Diagnostics, Mannheim, Germany) and a LightCycler (Roche Diagnostics). The primer pairs were 5′-AACATGTCCGAGAGGGAAGTGT-3′ and 5′-TATTTGCATTTCTTTGACTTCCATT-3′ for *Eed* and 5′-CAGGGTGTGATGGTGGGAATGGGTCAGAAG-3′ and 5′-TACGTACATGGCTGGGGTGTTGAAGGTCTC-3′ for *β-actin*. The amplification conditions for *Eed* were 95 °C for 10 min for one cycle, followed by 40 cycles of 95 °C denaturation for 10 s, 52 °C annealing for 5 s and 72 °C extension for 10 s. The same PCR conditions were used for *β-actin* except that the annealing temperature was 55 °C and the extension time was 20 s. The quantity of each transcript was measured from a standard curve, and the amounts of *Eed* transcript were normalized to *β-*actin transcript levels.

### Immunostaining

Cells grown on coverslips were fixed with 4% paraformaldehyde (Electron Microscopy Sciences, Hatfield, PA, USA) in 250 mM HEPES (pH 7.4) for 10 min, permeabilized with 1% Triton-X (Nacalai Tesque, Kyoto, Japan) in phosphate-buffered saline (PBS) for 20 min, and subjected to blocking with 100% Blocking One-P (Nacalai Tesque) for 20 min at room temperature. Cells were incubated in 2 μg/ml Cy3-conjugated anti-H3K27me3 antibody (CMA323)^63^ and 10 ng/ml DAPI (Thermo Fisher Scientific) in PBS containing 10% Blocking One-P and 0.5% Triton-X for 2 h at room temperature. After washing three times with PBS, coverslips were mounted using Prolong-Gold (Thermo Fisher Scientific).

### Library construction for the sequencing of vector insertion sites

Oligonucleotide sequences of linker DNA, PCR primers and sequencing primers are shown in Supplementary Table S11. For sequencing with Roche GS FLX, we digested 10 μg of genomic DNAs with *Hae*III and ligated it with splinkerette linker DNA^64^. In case ESCs were not selected with G418, it is highly likely that transfected plasmid DNAs were co-purified with genomic DNAs because genomic DNAs were extracted only 3 days after transfection. We therefore digested linker-ligated DNAs with *Dpn*I that cleaves Dam-methylated GATC site to avoid PCR-amplification of plasmid-derived DNAs. The junction between the vector DNA and flanking genomic region was amplified by nested PCR using vector-specific primers and linker-specific primers. To avoid reduction of PCR-amplification efficiency by overloading template DNA per reaction, we divided 10 μg of the splinkerette-ligated DNA into 8 tubes and conducted the first PCR reaction separately. The first PCR products were pooled from 8 tubes into one tube, mixed well, and an aliquot of the first PCR product was used as a template for the second PCR reaction. We observed that amplification of the transposon vector backbone derived from transposase-independent vector insertions markedly inhibited amplification of the transposon-inserted genomic regions. We also observed amplification of the internal region of the MLV vector because the MLV-specific primers anneal to both upstream and downstream LTRs. To avoid amplification of the vector backbone of the transposon and the MLV-internal regions, we cleaved splinkerette-ligated DNA and the first PCR products using the following enzymes prior to PCR reaction: *Pvu*II for MLV, *Xba*I for Tol2, *Dra*I and *Pfl*MI for PB, and *Bam*HI for SB. PCR products were size-fractionated by agarose gel electrophoresis, and 240–800-bp fragments were purified and sequenced by Roche GS FLX. For sequencing by Illumina GA2, we fragmented 10 μg genomic DNAs by ultrasound using Covaris S220 (Covaris, Woburn, MA, USA), blunt-ended, and ligated splinkerette linker DNA. Linker-ligated DNAs were digested with *Dpn*I to avoid PCR-amplification of transfected plasmid DNAs. The first PCR products were purified with streptavidin-coupled Dynabeads (Thermo Fisher Scientific) according to the manufacture’s protocol, and an aliquot of the purified DNA was used as a template for the second PCR reaction. PCR products were size-fractionated (300–400-bp) by agarose gel electrophoresis and 76-bp regions were sequenced at each end by Illumina GA2.

### Detection of insertion sites from Roche GS FLX sequence reads

The insertion sites of four vector types were determined by aligning the flanking sequence of the vector-tagging site against a mouse reference genome. Briefly, we removed the vector sequence from Roche GS FLX sequence reads using cross_match (http://www.phrap.org), and these trimmed reads with read length >25 nt were aligned against the mouse genome assembly (UCSC mm8) using BLAT^65^. Reads aligned to the reference genome with >90% identity and >90% coverage were extracted as candidate insertion loci. We confirmed the presence of specific motifs (such as TATA site for PB and TA site for SB) at the vector insertion sites. Reads with multiple alignment results were excluded from the analysis. Genomic coordinates of the insertion sites are deposited in the DDBJ database under the accession number DRA004513 (Analysis: DRZ007718 - DRZ007723).

### Detection of insertion sites from Illumina GA2 sequence reads

Insertion sites of Tol2 and PB were determined by aligning the flanking sequence of the vector region against the mouse reference genome. We trimmed off the vector sequences and linker sequences from Illumina GA2 sequence reads using cutadapt version 1.9.1^66^. For Tol2, reads with sequence mean base quality >30 were selected using Trimmomatic version 0.33^67^. This process was not conducted for PB because the presence of the consensus target sequence at the insertion sites could be used for sequence-quality check as described later. We discarded <25 bp reads to achieve high quality mapping. We used bwa-backtrack version 0.7.13^68^ to align the sequence reads against the mm8 reference assembly. We used ‘bwa aln’ and ‘bwa sampe -a 600’ options, and obtained chromosomal coordinates of the integration sites. We identified the reads mapped in proper pairs and uniquely aligned to the genome with high-mapping quality (>30) using samtools version 1.3^69^ (‘samtools view -f 66 -F 256’ option and awk command). For PB, we identified the reads with the consensus sequence of PB insertion. According to our experimental protocol, most of the insertion site sequences should be duplicated by PCR. Therefore, we used alignments supported by two or more reads using BEDTools version v2.16.2^70^ ‘genomecov command’, resulting in the final dataset of vector insertion sites. Genomic coordinates of the insertion sites are deposited in the DDBJ database under the accession number DRA002594 (Analysis: DRZ007730, DRZ007732, DRZ007734, DRZ007736).

### Control insertion site

To determine the characteristics of genomic distribution of the insertion sites, we created size-adjusted *in silico* control insertions^71^. For the Roche GS FLX sequencing datasets, we created control insertion sites by checking three criteria: distance from enzyme cutting sites, distance from insert motif (SB = TA, PB = TTAA), and presence/absence of secondary enzyme cut sites. First, we determined the enzyme cut sites (*Hae*III) on the mouse reference genome and randomly selected these positions as *in silico* enzyme cut sites. We created the insertion sites with matched fragment size and matched strand directionality relative to experimentally obtained vector insertion sites. Then, we selected the nucleotide position of specific motifs for vectors with specific insertion motif sequences. The difference of control fragment size from the observed fragment size must be less than 20-bp. Finally, we removed insertion sites containing secondary enzyme cut sites within its fragment (*Pvu*II for MLV, *Xba*I for Tol2, *Dra*I and *Pfl*MI for PB, and *Bam*HI for SB).

For the Illumina GA2 sequence dataset, we did not use the restriction enzymes for the genomic DNA fragmentation, and thus we did not apply the steps for enzyme cut sites. Specifically, for PB, we determined all genomic positions of the consensus sequence of PB insertion (TTAA) in the mm8 reference genome using Bowtie version 1.1.2^72^ ‘bowtie -a -v 0’ and randomly selected these positions. For Tol2, we randomly selected genomic positions from the mm8 reference genome using BEDTools version v2.16.2^70^ ‘random’ command because Tol2 does not have a consensus sequence for insertion. We then generated control insertion fragments having matched fragment sizes relative to the experimental dataset.

For both Roche GS FLX sequence dataset and Illumina GA2 dataset, we tested the mappability of these control insertion sites by aligning them using BWA (version 0.7.13). We aligned *in silico* matched control reads against the mm8 reference genome using bwa ‘aln’ and ‘samse’ with default setting, and identified the ones that were aligned to the predicted position and had high mapping quality (>30). We repeated this process 1,000 times, and obtained 1,000 datasets of *in silico* control insertion sites. Genomic coordinates of the matched control insertion sites for the Roche GS FLX and the Illumina GA2 sequence datasets are deposited in the DDBJ database under the accession numbers DRA004513 (Analysis: DRZ007745 - DRZ007750) and DRA002594 (Analysis: DRZ007741 - DRZ007744), respectively.

### Hotspot insertion site analysis

To compare the number of common insertion sites between different vectors with different numbers of vector insertions, we sampled insertion sites from both the observed and *in silico* control datasets by permutation. Specifically, we sampled 4,000 insertion sites from each vector dataset and its control dataset, and then we counted the number of 2 hit (2 insertions within 30-kb), 3 hit (3 insertions within 50-kb), and 4 hit (4 or more insertions within 100-kb) hotspot insertion loci. We repeated this random sampling from each dataset 1,000 times and calculated the mean of 2 hit, 3 hit, and 4 hit loci. We also performed the same hotspot insertion site analysis for the matched control datasets.

### Genome-wide distribution of insertions sites

To visualize the genome-wide distribution of insertion sites, we sampled 4,000 insertion sites from each vector dataset by permutation. We repeated this sampling 1,000 times, and we calculated the mean count of insertion sites in every 1-Mbp window. We performed this analysis for both vector inserts and their matched control dataset.

### Enrichment of insertion sites in exon-rich regions

We tested whether the insertion sites were enriched in exon-rich regions by dividing the genome into 500-kb bins (5,263 bins). First, we calculated the total size of exons in each bin and calculated the exon density (total exon size bp/500-kb). Then, we ranked these bins into five equal sized groups (exon density: lowest, low, mid, high, and highest). We determined the number of insertion sites located in each group for observed insertion sites and control insertion sites. The fractions of observed insertion sites located in bins with the highest exon density ranged from 38% (SB) to 54% (MLV). The fraction of control insertion sites located in bins with the highest exon density ranged from 22% to 26%.

### Inter-insertion distance calculation

We determined how closely insertion sites were clustered in each vector dataset by measuring the distance between insertion sites. Briefly, we sampled 4,000 insertions sites from each vector dataset 1,000 times by permutation. For each sampled dataset, we sorted the insertion sites by their chromosome coordinates. We measured the distance between (i + 1)^th^ insertions site and i^th^ insertion site as the inter-insertion distance. We calculated the descriptive statistics such as means and standard deviation of log10 transformed inter-insertion distance.

### Gene expression ranking and insertion sites

To compare the gene expression level with the frequency of insertion sites, we used microarray gene expression data from mouse ESCs^19^. We sorted genes based on the expression levels and divided them into eight equal sized bins. For example, the top ranked bin (bin #1) contained genes with the top 12.5 percentile expression levels. We counted the number of insertions located within 50-kb from genes and calculated the frequency of insertions in each bin using the following equation: (number of insertion in genes in a given bin)/(total number of insertion in all bins). We plotted the ratio of the observed rate/control rate. We performed similar analyses for insertion sites located inside genes.

### Genes and transcription start site analysis

We determined the relative location of various insertion sites to known genes using the RefSeq database. We compared our insertion sites to the NCBI RefSeq database and determined the fraction of insertion events located inside the RefSeq genes. The rate of in-gene insertion events in observed cases was divided by the rate of in-gene insertion events in *in silico* matched control cases. We also determined the frequency of insertion events relative to transcription start sites (TSSs) as reported by the RefSeq database. We used two approaches, gene-size scaled bins and 100-bp bin. In the first approach, we divided the insertion distances from the nearest TSSs by the size of targeted genes. In the second approach, we divided ± 5-kb TSS regions into 100-bp window bins, and we counted the number of cases inserted into each bin. The counts were divided by the total number of insertion events.

### Gene Set Enrichment Analysis

We performed Gene Set Enrichment Analysis^22^ using genes with frequent vector insertion sites. First, we collected genes with 3, 4, 5 or more insertion sites within ± 50-kb window for each vector dataset. Then, we compared the enrichment of these genes in gene expression patterns between two cell types, ESCs and neural progenitor cells (NPCs), using the microarray gene expression dataset reported by Mikkelsen *et al*. (GEO: GSM198062-GSM1198067)^19^. We used the software developed at the Broad Institute^22^ for analysis and used the normalized enrichment score (NES) as an indicator for the enrichment of frequently inserted genes.

### Relationship between insertion sites and distribution patterns of transcription regulators, histone modifications, DNase I hypersensitive sites and enhancer regions

We compared the distribution patterns of transcription regulators and histone modifications around vector insertion sites using the ChIP-seq dataset. To analyse the relationship between insertion sites and histone modifications, we used the ChIP-seq data from ESCs such as H3K4me3, H3K27me3, and H3K36me3 as reported by Mikkelsen *et al*. (NCBI GEO GSE12241)^19^. Specifically, we determined the mean ChIP-seq density values every 25-bp throughout ± 5-kb regions around the vector insertion sites (400 data points for each insertion site). We also used previously reported datasets of the histone modification-enriched regions (Hidden Markov Model^19^) to calculate the overlapping regions between H3K4me3 and H3K27me3. Furthermore, we determined the relationship between insertion sites and transcription regulators using the previously reported ChIP-seq dataset (GSM937540 for Brd4, GSM594600 and GSM594601 for P300^73^, and GSE22562^26^ for other transcription regulators). To visualize the distribution of transcription factor binding sites around insertion site, we calculated the relative distances to peak regions of transcription factor binding sites from each insertion sites within a ± 5-kb window. We analysed the correlation between insertion sites and DNase I hypersensitive sites using the ENCODE dataset of mouse ESCs on UCSC genome browser (GSM1014154). DNaseI sensitive zones were identified using the HotSpot algorithm^74^. Because the ENCODE dataset was based on the mouse mm9 genome assembly, we converted the coordinate of insertion sites based on mm8 genome assembly to the coordinates in mm9 genome assembly using the coordinate conversion tool, liftOver, from UCSC. We analysed the association between insertion sites and enhancers or super-enhancers by counting the number of insertion sites located inside these regions. We used the coordinates of enhancer and super-enhancer regions in mouse ESCs from Whyte *et al*.^20^. We determined the enrichment of enhancers in the observed insertions compared to those of the matched control insertions.

### Statistical Analysis

We performed statistical comparisons of the numbers of insertion sites located in genes, histone-modified regions, and the binding sites of transcriptional regulators between observed insertion sites and control insertion sites using binomial statistics. We estimated the probability of success using control datasets and calculated the significance. We applied FDR multiple testing correction to *P*-values. For the comparison of the insertion preference between different vectors or between *Wt* and *Eed*^*m/m*^ ESCs, we used Fisher’s exact test. For example, we compared the number of insertion sites located inside of ChIP-seq peaks for transcription factor binding sites between MLV and PB. We performed the comparison for six combinations (among four vectors) and adjusted the *P*-values for multiple testing using the FDR method. To compare sampled datasets, we used a bootstrap approach^36^. Specifically, we compared the number of hotspots and inter-insertion distances within each vector (observed vs. control) and between different vectors by sampling 4,000 insertion sites repeatedly. We created fragment-size matched and enzyme restriction site matched control insertion sites for each vector 1,000 times. We counted the number of control datasets with a mean value more extreme than the value observed in the experimental data 1,000 times. For example, we counted the number of insertion sites with <10 kb inter-insertion distance in the experimental dataset and in the matched control dataset. We counted the number of control sets with more insertion sites located inside RefSeq genes than the corresponding experimental dataset. The sum divided by 1,000 was the *P*-value, and this was adjusted for multiple testing using the FDR method. The significance threshold was *P* = 0.05.

### Southern blot analysis

Genomic DNAs of five G418-resistant and five G418-sensitive clones were digested by *Hin*dIII (MLV, PB, Tol2) or *Bgl*II (SB), separated by agarose gel, transferred to a nylon membrane, and hybridized with the neo probe using the standard protocol.

### **Accession codes:**

 Raw sequence reads, and genomic coordinates (mm8 assembly) for vector insertion sites and matched controls are deposited in the DDBJ database. Accession numbers are as follows.Roche FLX

- Sequence dataset: DRA004513.
- Genomic coordinates for vector insertion sites: DRA004513/Analysis: DRZ007718 (Tol2, +G418), DRZ007719 (MLV, +G418), DRZ007720 (PB, +G418), DRZ007721 (SB, +G418), DRZ007722 (MLV, −G418), DRZ007723 (PB, −G418).
- Genomic coordinates for matched controls: DRA004513/Analysis: DRZ007745 (Tol2, +G418), DRZ007746 (MLV, +G418), DRZ007747 (PB, +G418), DRZ007748 (SB, +G418), DRZ007749 (MLV, −G418), DRZ007750 (PB, −G418).

Illumina GA2

- Sequence dataset: DRX021632 (*Wt* ESCs, Tol2), DRX021634 (*Eed*^*m/m*^ ESCs, Tol2), DRX021636 (*Wt* ESCs, PB), DRX021638 (*Eed*^*m/m*^ ESCs, PB).
- Genomic coordinates for vector insertion sites: DRA002594/Analysis: DRZ007730 (*Wt* ESCs, Tol2), DRZ007732 (*Eed*^*m/m*^ ESCs, Tol2), DRZ007734 (*Wt* ESCs, PB), DRZ007736 (*Eed*^*m/m*^ ESCs, PB).
- Genomic coordinates for matched controls: DRA002594/Analysis: DRZ007741 (*Wt* ESCs, Tol2), DRZ007742 (*Eed*^*m/m*^ ESCs, Tol2), DRZ007743 (*Wt* ESCs, PB), DRZ007744 (*Eed*^*m/m*^ ESCs, PB).

## Additional Information

**How to cite this article:** Yoshida, J. *et al*. Chromatin states shape insertion profiles of the piggyBac, Tol2 and Sleeping Beauty transposons and murine leukemia virus. *Sci. Rep.* **7**, 43613; doi: 10.1038/srep43613 (2017).

**Publisher's note:** Springer Nature remains neutral with regard to jurisdictional claims in published maps and institutional affiliations.

## Supplementary Material

Supplementary Information
