# Supplementary material for: Chromatin states shape insertion profiles of the piggyBac, Tol2 and Sleeping Beauty transposons and murine leukemia virus
Source: Sci Rep. 2017 Mar 2;7:43613. doi: 10.1038/srep43613 (PMC5333637; doi:10.1038/srep43613)
Supplement: Supplementary Information [file srep43613-s1.pdf]

Supplementary Information

Chromatin states shape insertion profiles of the piggyBac, Tol2 and Sleeping Beauty transposons and murine leukemia virus

Junko Yoshida, Keiko Akagi, Ryo Misawa, Chikara Kokubu, Junji Takeda and Kyoji Horie

Supplementary Table S1. Consideration for the determination of vector insertion sites.

Supplementary Table S2. Processing of Roche GS FLX reads and the number of mapped loci.

Supplementary Table S3. Processing of Illumina GA2 reads and the number of mapped loci.

Supplementary Table S4. Vector comparison of inter-insertion distance.

Supplementary Table S5. Vector comparison of hotspot insertion frequency.

Supplementary Table S6. Vector comparison of enrichment inside the highest expressed genes.

Supplementary Table S7. Vector comparison of insertions into enhancer and super-enhancer regions.

Supplementary Table S8. Vector comparison of insertions at histone-modified regions.

Supplementary Table S9. Vector comparison of insertions at the binding sites of regulatory proteins.

Supplementary Table S10. Comparison of the findings between previous studies and the current study.

Supplementary Table S11. Oligonucleotides for splinkerette-PCR and sequencing of vector insertion sites.

Supplementary Fig. S1. Copy number of vector DNA in G418-resistant and -sensitive clones.

Supplementary Fig. S2. Genome-wide distribution of vector insertion sites.

Supplementary Fig. S3. Grouping of genomic regions by exon density.

Supplementary Fig. S4. UCSC genome browser view of a PB insertion hotspot.

Supplementary Fig. S5. Comparison between with and without G418 selection analysed with Roche GS FLX.

Supplementary Fig. S6. Insertion preference of PB and Tol2 analyzed with Illumina GA2.

Supplementary Table S1. Consideration for the determination of vector insertion sites

Factors contributing to bias	Solution
1. Small dataset size	High-throughput sequencing<br>Roche GS FLX, Illumina GA2
2. Transcriptional silencing of the neo gene	Multiple vector insertion<br>No G418 selection
3. Local hopping of DNA transposons	Independent transfections
4. Consensus target sequence<br>piggyBac: TTAA<br>Sleeping Beauty: TA	*In silico* control
5. Distribution of restriction sites	*In silico* control<br>Sonication

Supplementary Table S2. Processing of Roche GS FLX reads and the number of mapped loci

Vector	Expt.[a]	G418 selection	Raw reads	Processed reads[b]				Mapped loci[c]	
			count	count	%valid	mean size (bp)	stddev	count	loci/processed reads
MLV	1	+	3,998	3,179	79.51%	67.02	23.75	1,673	52.63%
	2	+	2,188	1,870	85.47%	67.07	20.34	1,090	58.29%
	3	+	9,352	6,594	70.51%	67.83	24.01	3,131	47.48%
Total			15,538	11,643	74.93%			5,894	50.62%
PB	1	+	6,091	4,809	78.95%	117.62	66.76	2,194	45.62%
	2	+	7,794	5,473	70.22%	119.85	68.57	2,464	45.02%
	3	+	4,530	3,605	79.58%	125.27	68.67	1,710	47.43%
Total			18,415	13,887	75.41%			6,368	45.86%
Tol2	1	+	4,518	2,777	61.47%	111.02	48.25	1,929	69.46%
	2	+	4,804	2,433	50.65%	90.38	43.29	1,624	66.75%
	3	+	2,056	1,268	61.67%	98.73	43.08	886	69.87%
Total			11,378	6,478	56.93%			4,439	68.52%
SB	1	+	6,941	5,425	78.16%	134.55	73.6	3,040	56.04%
	2	+	5,863	4,543	77.49%	134.97	70.21	2,603	57.30%
	3	+	6,514	5,241	80.46%	135.94	70.54	2,998	57.20%
Total			19,318	15,209	78.73%			8,641	56.82%
MLV	1	-	7,511	5,201	69.25%	158.52	84.31	2,072	39.84%
	2	-	7,729	4,982	64.46%	160.73	84.96	2,053	41.21%
	3	-	6,167	4,077	66.11%	156.05	79.89	1,705	41.82%
Total			21,407	14,260	66.61%			5,830	40.88%
PB	1	-	7,317	4,389	59.98%	140.25	91.08	1,536	35.00%
	2	-	9,699	4,366	45.01%	155.42	97.59	1,285	29.43%
	3	-	9,516	4,786	50.29%	144.88	94.56	1,538	32.14%
Total			26,532	13,541	51.04%			4,359	32.19%

[a] Expt. 1-3 indicate three independent transfections.

[b] Vector- and adaptor-derived sequences were trimmed.

[c] Mapping was conducted by BLAT search using mm8 mouse genome assembly as a reference. In case multiple reads were mapped at the same coordinate, this was regarded as a single insertion event.

Supplementary Table S3. Processing of Illumina GA2 reads and the number of mapped loci

Vector	Cell type	Raw reads	Processed reads[a]	Aligned reads[b] (Q>30)	Mapped loci[c]
Tol2	*Wt*	24,999,311	7,427,316	4,839,750	6,594
PB	*Wt*	33,347,781	12,493,839	2,796,459	3,261
Tol2	$Eed^{m/m}$	25,284,729	737,563	98,555	3,016
PB	$Eed^{m/m}$	27,369,650	1,307,017	601,726	2,868

[a]Vector and linker sequences were trimmed off . For Tol2, reads with sequence mean base quality > 30 were selected using Trimmomatic. This process was not conducted for PB because the presence of the consensus target sequence (TTAA) at the insertion sites could be used for sequence-quality checks in the downstream analysis.
[b]Reads were aligned by BWA aligner against mouse genome assembly mm8, and only the reads with alignment quality > 30 were used for further analysis. For PB, only the reads with the consensus target sequence were selected.
[c]Because most of the insertion site sequences should be duplicated by PCR, we used alignments supported by two or more reads using BEDTools. Multiple reads mapped at the same coordinate were regarded as a single insertion event.

Supplementary Table S4. Vector comparison of inter-insertion distance

Vector1	Fraction of inter-insertion distance < 10kb (vector1)	Vector2	Fraction of inter-insertion distance < 10kb (vector2)	Ratio	Number of trials	Number of cases of vector1 < vector2	*P*-value	Significance
MLV	16.93%	PB	5.71%	2.97	1000	0	<0.001	*
MLV	16.93%	Tol2	4.95%	3.42	1000	0	<0.001	*
MLV	16.93%	SB	2.58%	6.57	1000	0	<0.001	*
PB	5.71%	Tol2	4.95%	1.15	1000	0	<0.001	*
PB	5.71%	SB	2.58%	2.22	1000	0	<0.001	*
Tol2	4.95%	SB	2.58%	1.92	1000	0	<0.001	*

P-values were calculated by bootstrapping.

* $P<0.05$.

Supplementary Table S5. Vector comparison of hotspot insertion frequency

Hit type	Vector1	Mean number of hotspots in vector1	Vector2	Mean number of hotspots in vector2	Ratio (vector1/ vector2)	Number of trials	Number of cases of vector1 < vector2	*P*-value	Adjusted *P*-value	Significance
2-hits	MLV	790.23	PB	427.58	1.85	1000	0	<0.001	<0.001	*
2-hits	MLV	790.23	Tol2	415.96	1.90	1000	0	<0.001	<0.001	*
2-hits	MLV	790.23	SB	273.98	2.88	1000	0	<0.001	<0.001	*
2-hits	PB	427.58	Tol2	415.96	1.03	1000	52	0.052	0.052	
2-hits	PB	427.58	SB	273.98	1.56	1000	0	<0.001	<0.001	*
2-hits	Tol2	415.96	SB	273.98	1.52	1000	0	<0.001	<0.001	*
3-hits	MLV	293.80	PB	96.31	3.05	1000	0	<0.001	<0.001	*
3-hits	MLV	293.80	Tol2	75.27	3.90	1000	0	<0.001	<0.001	*
3-hits	MLV	293.80	SB	31.03	9.47	1000	0	<0.001	<0.001	*
3-hits	PB	96.31	Tol2	75.27	1.28	1000	0	<0.001	<0.001	*
3-hits	PB	96.31	SB	31.03	3.10	1000	0	<0.001	<0.001	*
3-hits	Tol2	75.27	SB	31.03	2.43	1000	0	<0.001	<0.001	*
4-hits or more	MLV	171.73	PB	43.61	3.94	1000	0	<0.001	<0.001	*
4-hits or more	MLV	171.73	Tol2	38.18	4.50	1000	0	<0.001	<0.001	*
4-hits or more	MLV	171.73	SB	12.67	13.55	1000	0	<0.001	<0.001	*
4-hits or more	PB	43.61	Tol2	38.18	1.14	1000	25	0.025	0.026	*
4-hits or more	PB	43.61	SB	12.67	3.44	1000	0	<0.001	<0.001	*
4-hits or more	Tol2	38.18	SB	12.67	3.01	1000	0	<0.001	<0.001	*

P-values were calculated by bootstrapping and adjusted by FDR for multiple comparisons.
*$P<0.05$.

Supplementary Table S6. Vector comparison of enrichment inside the highest expressed genes

Vector1	Vector2	Fraction of insertions (vector1)	Fraction of insertions (vector2)	*P*-value	Adjusted *P*-value	Significance
MLV	PB	13.56%	14.95%	1.51E-01	1.51E-01	
MLV	Tol2	13.56%	8.76%	1.85E-07	3.70E-07	*
MLV	SB	13.56%	9.92%	6.55E-06	9.83E-06	*
PB	Tol2	14.95%	8.76%	2.08E-11	1.25E-10	*
PB	SB	14.95%	9.92%	2.78E-10	8.33E-10	*
Tol2	SB	8.76%	9.92%	1.47E-01	1.51E-01	

P-values were calculated by Fisher's exact test and adjusted by FDR for multiple comparisons.

*$P<0.05$.

Supplementary Table S7. Vector comparison of insertions into enhancer and super-enhancer regions

Type	Vector1	Fraction of insertions (vector1)	Vector2	Fraction of insertions (vector2)	Ratio	*P*-value	Adjusted *P*-value	Significance
Enhancer	MLV	9.65%	PB	6.27%	1.54	4.26E-12	5.12E-12	*
Enhancer	MLV	9.65%	Tol2	2.12%	4.56	7.64E-61	1.53E-60	*
Enhancer	MLV	9.65%	SB	0.84%	11.43	6.03E-149	3.62E-148	*
Enhancer	PB	6.27%	Tol2	2.12%	2.96	2.06E-26	3.09E-26	*
Enhancer	PB	6.27%	SB	0.84%	7.42	5.98E-82	1.79E-81	*
Enhancer	Tol2	2.12%	SB	0.84%	2.51	2.89E-09	2.89E-09	*
Super-enhancer	MLV	2.65%	PB	1.52%	1.74	1.38E-05	1.66E-05	*
Super-enhancer	MLV	2.65%	Tol2	0.41%	6.53	3.26E-21	6.52E-21	*
Super-enhancer	MLV	2.65%	SB	0.08%	32.67	3.17E-52	1.90E-51	*
Super-enhancer	PB	1.52%	Tol2	0.41%	3.76	4.82E-09	7.23E-09	*
Super-enhancer	PB	1.52%	SB	0.08%	18.80	2.45E-28	7.35E-28	*
Super-enhancer	Tol2	0.41%	SB	0.08%	5.01	1.45E-04	1.45E-04	*

P-values were calculated by Fisher's exact test and adjusted by FDR for multiple comparisons.

*$P<0.05$.

Supplementary Table S8. Vector comparison of insertions at histone-modified regions

Histone modification	Vector1	Fraction of insertions (vector1)	Vector2	Fraction of insertions (vector2)	Ratio	*P*-value	Adjusted *P*-value	Significance
H3K4me3	MLV	14.40%	PB	9.74%	1.48	1.84E-015	2.21E-015	*
H3K4me3	MLV	14.40%	Tol2	9.17%	1.57	3.76E-016	5.64E-016	*
H3K4me3	MLV	14.40%	SB	1.02%	14.14	1.14E-242	6.82E-242	*
H3K4me3	PB	9.74%	Tol2	9.17%	1.06	3.34E-001	3.34E-001	
H3K4me3	PB	9.74%	SB	1.02%	9.56	8.46E-145	2.54E-144	*
H3K4me3	Tol2	9.17%	SB	1.02%	9.00	1.11E-112	2.22E-112	*
H3K27me3	MLV	1.43%	PB	1.65%	0.86	3.40E-001	4.08E-001	
H3K27me3	MLV	1.43%	Tol2	3.51%	0.41	5.01E-012	1.50E-011	*
H3K27me3	MLV	1.43%	SB	1.39%	1.03	8.86E-001	8.86E-001	
H3K27me3	PB	1.65%	Tol2	3.51%	0.47	9.42E-010	1.88E-009	*
H3K27me3	PB	1.65%	SB	1.39%	1.19	1.97E-001	2.96E-001	
H3K27me3	Tol2	3.51%	SB	1.39%	2.53	1.09E-014	6.56E-014	*
H3K4me3 + H3K27me3	MLV	0.64%	PB	0.47%	1.37	2.24E-001	2.24E-001	
H3K4me3 + H3K27me3	MLV	0.64%	Tol2	1.73%	0.37	2.06E-007	4.12E-007	*
H3K4me3 + H3K27me3	MLV	0.64%	SB	0.24%	2.65	2.67E-004	4.00E-004	*
H3K4me3 + H3K27me3	PB	0.47%	Tol2	1.73%	0.27	1.25E-010	3.74E-010	*
H3K4me3 + H3K27me3	PB	0.47%	SB	0.24%	1.94	2.24E-002	2.69E-002	*
H3K4me3 + H3K27me3	Tol2	1.73%	SB	0.24%	7.14	1.28E-019	7.69E-019	*

P-values were calculated by Fisher's exact test and adjusted by FDR for multiple comparisons.

*$P<0.05$.

Supplementary Table S9. Vector Comparison of insertions at the binding sites of regulatory proteins

Regulatory protein	Vector1	Fraction of insertions (vector1)	Vector2	Fraction of insertions (vector2)	Ratio	*P*-value	Adjusted *P*-value	Significance
Brd4	MLV	25.30%	PB	17.20%	1.47	4.23E-28	4.23E-28	*
Brd4	MLV	25.30%	Tol2	9.37%	2.70	1.05E-100	2.09E-100	*
Brd4	MLV	25.30%	SB	1.46%	17.35	0.00E+00	0.00E+00	*
Brd4	PB	17.20%	Tol2	9.37%	1.83	5.84E-32	7.01E-32	*
Brd4	PB	17.20%	SB	1.46%	11.79	1.29E-285	3.87E-285	*
Brd4	Tol2	9.37%	SB	1.46%	6.43	1.63E-96	2.44E-96	*
CTCF	MLV	0.92%	PB	3.13%	0.29	8.03E-19	2.41E-18	*
CTCF	MLV	0.92%	Tol2	2.86%	0.32	1.00E-13	1.51E-13	*
CTCF	MLV	0.92%	SB	0.94%	0.98	9.30E-01	9.30E-01	
CTCF	PB	3.13%	Tol2	2.86%	1.09	4.58E-01	5.49E-01	
CTCF	PB	3.13%	SB	0.94%	3.33	2.09E-22	1.25E-21	*
CTCF	Tol2	2.86%	SB	0.94%	3.05	9.35E-16	1.87E-15	*
Med12	MLV	3.34%	PB	3.41%	0.98	8.81E-01	8.81E-01	
Med12	MLV	3.34%	Tol2	1.49%	2.25	1.38E-09	1.65E-09	*
Med12	MLV	3.34%	SB	0.37%	9.03	1.44E-46	4.32E-46	*
Med12	PB	3.41%	Tol2	1.49%	2.29	2.71E-10	4.07E-10	*
Med12	PB	3.41%	SB	0.37%	9.20	1.79E-49	1.07E-48	*
Med12	Tol2	1.49%	SB	0.37%	4.01	1.86E-11	3.72E-11	*
Med1	MLV	3.94%	PB	3.91%	1.01	9.63E-01	9.63E-01	
Med1	MLV	3.94%	Tol2	1.69%	2.33	9.68E-12	1.16E-11	*
Med1	MLV	3.94%	SB	0.31%	12.60	3.09E-62	9.26E-62	*
Med1	PB	3.91%	Tol2	1.69%	2.31	7.76E-12	1.16E-11	*
Med1	PB	3.91%	SB	0.31%	12.51	1.27E-63	7.60E-63	*
Med1	Tol2	1.69%	SB	0.31%	5.41	3.05E-16	6.11E-16	*
Nanog	MLV	1.66%	PB	3.14%	0.53	1.03E-07	1.55E-07	*
Nanog	MLV	1.66%	Tol2	0.88%	1.89	4.85E-04	5.82E-04	*
Nanog	MLV	1.66%	SB	0.42%	3.99	2.46E-14	4.92E-14	*
Nanog	PB	3.14%	Tol2	0.88%	3.57	1.04E-16	3.13E-16	*
Nanog	PB	3.14%	SB	0.42%	7.54	1.50E-41	8.98E-41	*
Nanog	Tol2	0.88%	SB	0.42%	2.11	1.35E-03	1.35E-03	*
Nipbl	MLV	0.92%	PB	1.19%	0.77	1.58E-01	1.58E-01	
Nipbl	MLV	0.92%	Tol2	0.45%	2.03	6.34E-03	7.60E-03	*
Nipbl	MLV	0.92%	SB	0.09%	9.90	4.44E-14	1.33E-13	*
Nipbl	PB	1.19%	Tol2	0.45%	2.65	3.78E-05	7.55E-05	*
Nipbl	PB	1.19%	SB	0.09%	12.89	2.90E-20	1.74E-19	*
Nipbl	Tol2	0.45%	SB	0.09%	4.87	6.50E-05	9.75E-05	*
Oct4	MLV	1.41%	PB	2.91%	0.48	9.99E-09	1.50E-08	*
Oct4	MLV	1.41%	Tol2	1.01%	1.39	8.74E-02	8.74E-02	
Oct4	MLV	1.41%	SB	0.45%	3.12	1.18E-09	2.36E-09	*
Oct4	PB	2.91%	Tol2	1.01%	2.87	3.40E-12	1.02E-11	*
Oct4	PB	2.91%	SB	0.45%	6.44	2.13E-35	1.28E-34	*
Oct4	Tol2	1.01%	SB	0.45%	2.25	2.80E-04	3.36E-04	*
P300	MLV	4.09%	PB	4.44%	0.92	3.48E-01	3.48E-01	
P300	MLV	4.09%	Tol2	2.61%	1.56	4.27E-05	5.13E-05	*
P300	MLV	4.09%	SB	0.44%	9.30	1.26E-57	3.79E-57	*
P300	PB	4.44%	Tol2	2.61%	1.70	4.48E-07	6.71E-07	*
P300	PB	4.44%	SB	0.44%	10.11	8.64E-67	5.18E-66	*
P300	Tol2	2.61%	SB	0.44%	5.94	6.37E-26	1.27E-25	*
Pol2	MLV	14.88%	PB	10.10%	1.47	9.95E-16	9.95E-16	*
Pol2	MLV	14.88%	Tol2	5.11%	2.91	3.85E-61	7.70E-61	*
Pol2	MLV	14.88%	SB	0.94%	15.87	1.51E-259	9.09E-259	*
Pol2	PB	10.10%	Tol2	5.11%	1.97	8.58E-22	1.03E-21	*
Pol2	PB	10.10%	SB	0.94%	10.77	1.06E-157	3.19E-157	*
Pol2	Tol2	5.11%	SB	0.94%	5.46	4.59E-47	6.89E-47	*
Smc1	MLV	0.88%	PB	2.58%	0.34	3.31E-13	6.61E-13	*
Smc1	MLV	0.88%	Tol2	2.14%	0.41	1.29E-07	1.94E-07	*
Smc1	MLV	0.88%	SB	0.56%	1.59	2.41E-02	2.89E-02	*
Smc1	PB	2.58%	Tol2	2.14%	1.20	1.60E-01	1.60E-01	
Smc1	PB	2.58%	SB	0.56%	4.64	2.58E-25	1.55E-24	*
Smc1	Tol2	2.14%	SB	0.56%	3.85	1.91E-15	5.73E-15	*
Smc3	MLV	0.39%	PB	1.99%	0.20	2.67E-17	1.60E-16	*
Smc3	MLV	0.39%	Tol2	1.73%	0.22	6.00E-12	1.20E-11	*
Smc3	MLV	0.39%	SB	0.57%	0.69	1.50E-01	1.79E-01	
Smc3	PB	1.99%	Tol2	1.73%	1.15	3.51E-01	3.51E-01	
Smc3	PB	1.99%	SB	0.57%	3.52	1.35E-15	4.06E-15	*
Smc3	Tol2	1.73%	SB	0.57%	3.06	4.97E-10	7.46E-10	*
Sox2	MLV	1.51%	PB	2.83%	0.53	6.66E-07	9.99E-07	*
Sox2	MLV	1.51%	Tol2	0.88%	1.72	3.96E-03	3.96E-03	*
Sox2	MLV	1.51%	SB	0.37%	4.08	2.04E-13	4.08E-13	*
Sox2	PB	2.83%	Tol2	0.88%	3.22	1.73E-13	4.08E-13	*
Sox2	PB	2.83%	SB	0.37%	7.63	7.25E-38	4.35E-37	*
Sox2	Tol2	0.88%	SB	0.37%	2.37	3.52E-04	4.23E-04	*
TBP	MLV	1.54%	PB	1.95%	0.79	9.83E-02	1.18E-01	
TBP	MLV	1.54%	Tol2	1.49%	1.04	8.71E-01	8.71E-01	
TBP	MLV	1.54%	SB	0.17%	8.89	5.22E-22	1.57E-21	*
TBP	PB	1.95%	Tol2	1.49%	1.31	7.46E-02	1.12E-01	
TBP	PB	1.95%	SB	0.17%	11.22	5.43E-31	3.26E-30	*
TBP	Tol2	1.49%	SB	0.17%	8.57	1.51E-18	3.02E-18	*

P-values were calculated by Fisher's exact test and adjusted by FDR for multiple comparisons.

*P<0.05

Supplementary Table S10. Comparison of the findings between previous studies and the current study [a]

	Previous studies [b]				Current study [c]				
	MLV	PB	Tol2	SB	MLV	PB	Tol2	SB	Note
**Gene**	Preference for gene regions (42,43)	Preference for gene regions (33,38)	Preference for gene regions (31,38)	Preference for gene regions (33,38)	Correlation with genome-wide exon density; Preference for gene regions	Correlation with genome-wide exon density; Preference for gene regions	Correlation with genome-wide exon density; Preference for gene regions	Correlation with genome-wide exon density; Preference for gene regions	
**Insertion hotspot**	Half of the insertions were in <2% of the genomes (36)	NA	NA	NA	Strong	Medium	Medium	Low	
**Gene expression**	Strong preference (37)	Preference (33)	NA	Some preference (33)	Strong preference	Moderate preference	Weak preference	Weak preference	
**TSS**	Strong preference; Bimodal pattern [d] (34,37,43)	Preference (33,34,38)	Preference (31,35,38)	No preference (34,39)	Strong preference; Bimodal pattern [d]	Preference; Bimodal pattern [d]	Preference; Bimodal pattern [d]	No preference	
**Histone modifications**	Strong preference for active marks (34,36,37,41,42), especially for the regions enriched with multiple active modifications (36)	Preference for active modifications (33)	Inverse correlation with H3K27me3 in HeLa cells (31)	Minimum preference for active marks (33)	Strong preference for super-enhancer regions	Preference for super-enhancer regions	Preference for bivalent modification of H3K4me3 and H3K27me3	Minimum preference for histone modifications	The cell lines analysed for Tol2 were different between the previous study and the current study
**Developmentally regulated genes [e]**	NA	NA	NA	NA	Strong preference for ESC-specific genes	Preference for ESC-specific genes	Preference for inducible genes	Some preference for inducible genes	
**DNase I HS**	Preference (36); Bimodal pattern [f] (34)	Preference (17,33,38)	Preference (38)	No preference (38)	Preference; Bimodal pattern [f]	Preference; Single peak	Preference; Single narrow peak	No preference	
**Transcriptional regulators**	Preference for the binding sites of transcriptional regulators(34); Interaction with BET proteins (41)	Preference for the binding sites of transcriptional regulators(34); Interaction with BET proteins (34)	NA	Weak preference (34)	Strong preference for the binding sites of multiple transcriptional regulators; Bimodal pattern [f]	Preference for the binding sites of multiple transcriptional regulators; Single peak	Preference for the binding sites of transcriptional regulators; Association with ESC-specific transcription factors is weaker than PB	No preference	Cluster of various transcriptional regulators at the insertion sites is consistent with the preference for super-enhancer region
**Chromatin architectural proteins**	Preference for the binding sites of CTCF in primary human $CD4^+$ T cells (34), HepG2(36), K562 (36)	Preference for the binding sites of CTCF in primary human CD4+ T cells (34), and Smc1, Smc3, Med1, Med12 in mouse ESCs (33)	NA	No preference for Smc1, Smc3, Med1, Med12 in mouse ESCs (33)	No preference for CTCF; Preference for Smc1, Smc3, Med1, Med12, Nipbl	Preference for CTCF, Smc1, Smc3, Med1, Med12, Nipbl	Preference for CTCF, Smc1, Smc3, Med1, Med12, Nipbl	No preference for CTCF, Smc1, Smc3, Med1, Med12, Nipbl	
**Other features**	Some preference for nucleosomal DNA (34)	Local DNA flexibility (35)	Local DNA flexibility (35)	Preference for some repetitive elements (39,40) and zigzag pattern deformability of local DNA (32,44)	NA	NA	NA	NA	

[a] The numbers in parenthesis correspond to the reference number in the main text. NA, not applicable.

[b] Results obtained from various cells are summarized unless indicated.

[c] Red, new findings; Blue, similar observations were reported previously; however, comparison between the four vectors has not been done or analysis in ESCs has not been reported.

[d] Bimodal pattern indicates that the peak of the insertion was observed upstream and downstream of TSS.

[e] Genes differentially expressed between ESCs and NPCs.

[f] Bimodal pattern indicates that the peak of DNase I HS or enrichment of the binding sites of transcriptional regulators was observed upstream and downstream of the insertion site.

Supplementary Table S11. Oligonucleotides for splinkerette-PCR and sequencing of vector insertion sites

Usage	Primer name	Sequence
**For Roche GS FLX**		
Splinkerette	Spl-top	CGAATCGTAACCGTTCGTACGAGAATTCGTACGAGAATCGCTGTCCTCTCCAACGAGCCAAGG
	SplB-BLT	CCTTGGCTCGTTTTTTTTTGCAAAAA
1st nested-PCR primer		
MLV	T/DR	AGTGTATGTAAACTTCTGACCCACTGG
Tol2	L200-1	CTTTTTGACTGTAAATAAAATTGTAAGGAG
PB	PB5-P1	AAGCGGCGACTGAGATGTCCTAAATG
SB	SBR-P1	CTAACTGACCTAAGACAGGGAATTTTTAC
Splinkerette	Spl-P1	CGAATCGTAACCGTTCGTACGAGAA
2nd nested-PCR primer		
MLV	Bal-FLX1	CCATCTGTTCCCTCCCTGTCTCAGACTCTTGTGTCATGCACAAAGTAGATGTCC
	Bal-FLX2	CCATCTGTTCCCTCCCTGTCTCAGCAGCTTGTGTCATGCACAAAGTAGATGTCC
	Bal-FLX3	CCATCTGTTCCCTCCCTGTCTCAGTGACTTGTGTCATGCACAAAGTAGATGTCC
Tol2	L200-3-FLX1	CCATCTGTTCCCTCCCTGTCTCAGACTATAATACTTAAGTACAGTAATCAAG
	L200-3-FLX2	CCATCTGTTCCCTCCCTGTCTCAGCAGATAATACTTAAGTACAGTAATCAAG
	L200-3-FLX3	CCATCTGTTCCCTCCCTGTCTCAGTGAATAATACTTAAGTACAGTAATCAAG
PB	V-PB5-P3-FLX1	CCATCTCATCCCTGCGTGTCTCCGACTCAGACAGTGAAAGAGAGAGCAATATTTCAAGAATG
	V-PB5-P3-FLX2	CCATCTCATCCCTGCGTGTCTCCGACTCAGCATAGGAAAGAGAGAGCAATATTTCAAGAATG
	V-PB5-P3-FLX3	CCATCTCATCCCTGCGTGTCTCCGACTCAGTGTCAGAAAGAGAGAGCAATATTTCAAGAATG
SB	V-SBR-P3-FLX1	CCATCTCATCCCTGCGTGTCTCCGACTCAGACAGTAAAAGTGAGTTTAAATGTATTTGGCTAAGG
	V-SBR-P3-FLX2	CCATCTCATCCCTGCGTGTCTCCGACTCAGCATAGAAAAGTGAGTTTAAATGTATTTGGCTAAGG
	V-SBR-P3-FLX3	CCATCTCATCCCTGCGTGTCTCCGACTCAGTGTCAAAAAGTGAGTTTAAATGTATTTGGCTAAGG
Splinkerette	Spl-P2-FLX (for Tol2 & MLV)	CCTATCCCCTGTTGCGTGTCTCAGTCGTACGAGAATCGCTGTCCTCTCC
	V-Spl-P2-FLX (for PB & SB)	CCTATCCCCTGTGTGCCTTGGCAGTCTCAGTCGTACGAGAATCGCTGTCCTCTCC
**For Illumina GA2**		
Splinkerette	SPLK-A	CGAAGAGTAACCGTTGCTAGGAGAGACCGTGGCTGAATGAGACTGGTGTCGACACTAGTGG
	SPLK-BLT	CCACTAGTGTCGACACCAGTCTCTAATTTTTTTTTTTCAAAAAAA
1st nested-PCR primer		
Tol2	Bio-L200-1	Biotin-GACGACCTTTTTGACTGTAAATAAAATTGTAAGGAG
PB	Bio-PB5-P1	Biotin-GCAACTAAGCGGCGACTGAGATGTCCTAAATG
Splinkerette	Splink1	CGAAGAGTAACCGTTGCTAGGAGAGACC
2nd nested-PCR primer		
Tol2	P5-L200-4	AATGATACGGCGACCACCGAGATCTACACTCCCAAAAATAATACTTAAGTACAGTAATCAAG
PB	P5-PB5pr2	AATGATACGGCGACCACCGAGATCTACACTCATGCGTCAATTTTACGCAGACTATC
Splinkerette	P7-Splink2	CAAGCAGAAGACGGCATACGAGATGTGGCTGAATGAGACTGGTGTCGAC
Sequencing primer		
Tol2	L200seq1	ACTTAAGTACAGTAATCAAGTAAAATTACTCAAGTAC
PB	PB5seq	ATGCGTCAATTTTACGCAGACTATCTTTC
Splinkerette	Splink2seq	GTGGCTGAATGAGACTGGTGTCGAC

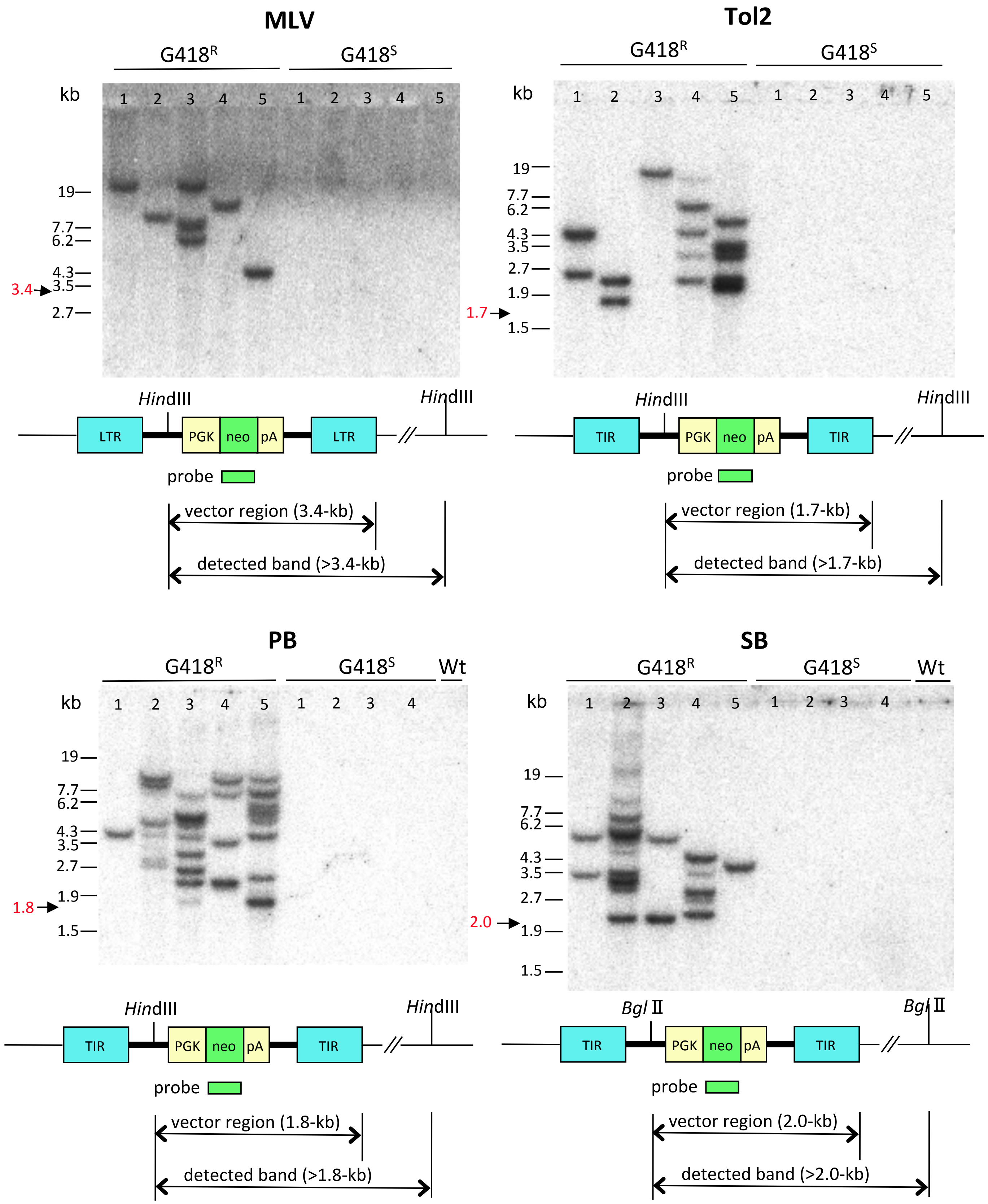

Supplementary Fig. S1. Copy number of vector DNA in G418-resistant and -sensitive clones
Copy number of vector DNA was examined by Southern blot analysis in five G418-resistant and four or five G418-sensitive clones for each vector. Each insertion site was detected as a different size of DNA fragment encompassing from the *Hin*dIII or *Bgl*II site within the vector DNA to a *Hin*dIII or *Bgl*II site in the flanking region. TIR, terminal inverted repeat; Wt, wild-type ESCs.

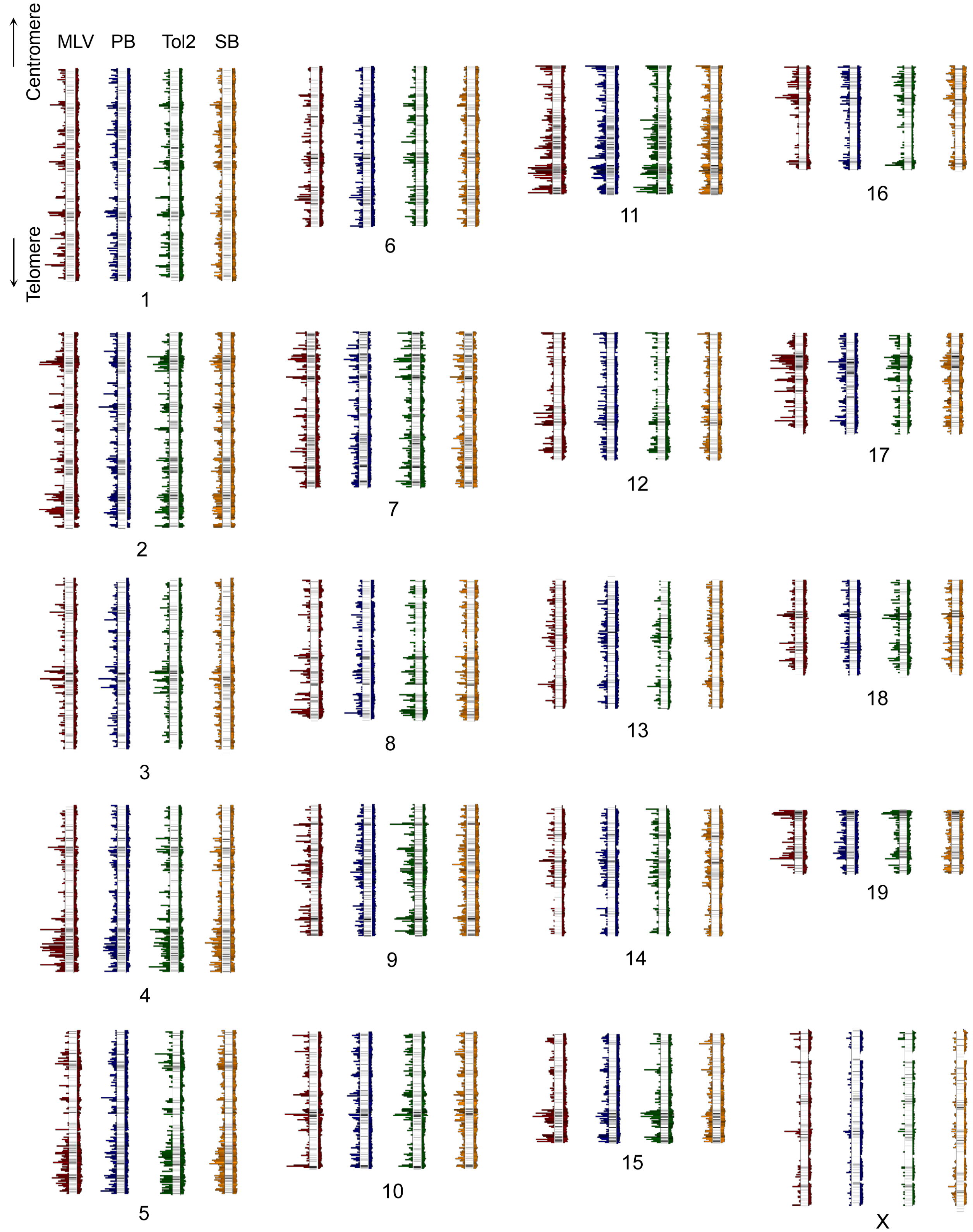

Supplementary Fig. S2. Genome-wide distribution of vector insertion sites

Each number indicates chromosome number. The result of chromosome 11 is same as in Fig. 2a. See Fig. 2a legend for details.

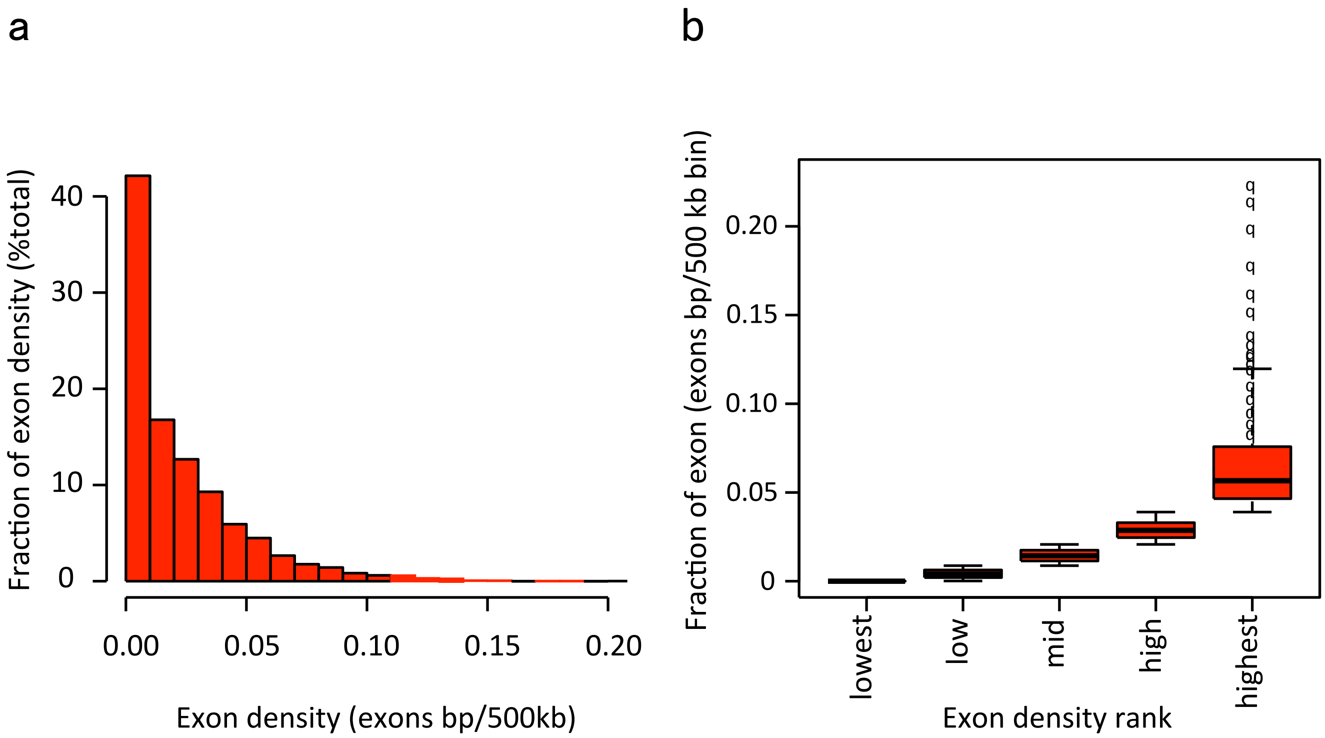

Supplementary Fig. S3. Grouping of genomic regions by exon density
(a) Histogram of exon density. Genomic regions were divided every 500-kb. Exon density in each 500-kb bin is shown on the X-axis and the corresponding frequency of each exon density is presented on the Y-axis.
(b) Distribution of exon density. Bins of 500-kb genomic regions described in (a) were divided into 5 equal sized groups.

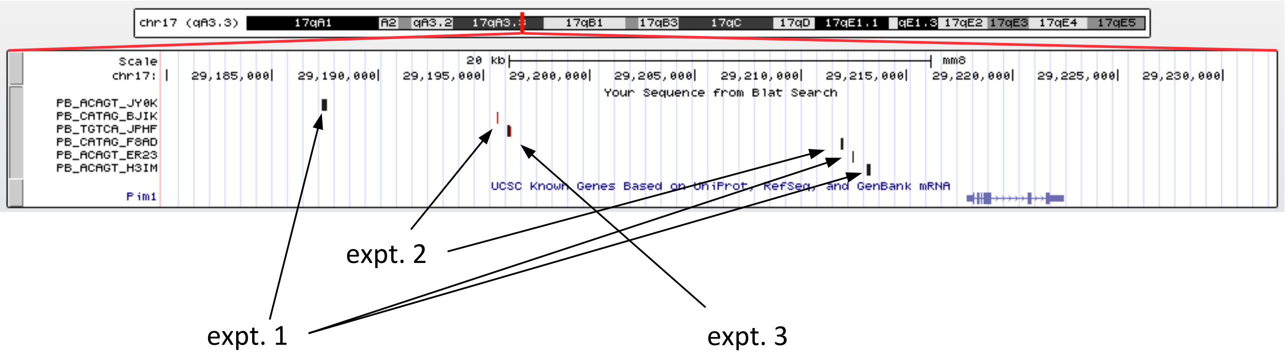

Supplementary Fig. S4. UCSC genome browser view of a PB insertion hotspot
Expt. 1 – 3 indicate three independent transfections.

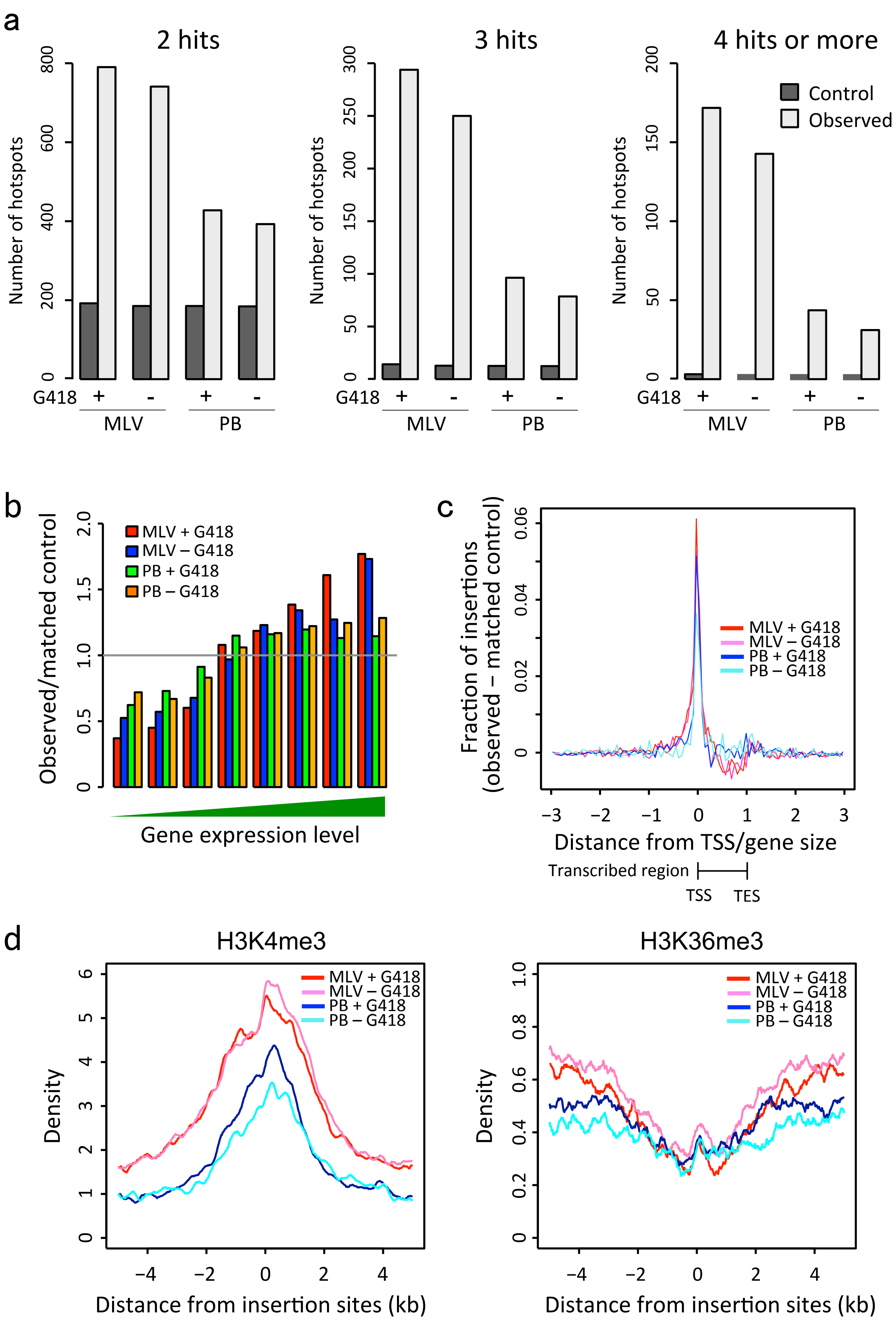

Supplementary Fig. S5. Comparison between with and without G418 selection analysed with Roche GS FLX.
(a) Hotspot insertion sites. Numbers of hotspots out of 4,000 insertion events are shown.
(b) Frequency of vector insertion nearby genes (± 50-kb) relative to their expression levels.
(c) Relative distance between TSS and vector insertion sites. TES, transcription end site.
(d) Correlation of insertion sites with histone modifications.
The results are similar between G418 selection and no G418 selection, indicating that silencing of the gene cassette is rare and does not affect the interpretation of the vector insertion preferences.

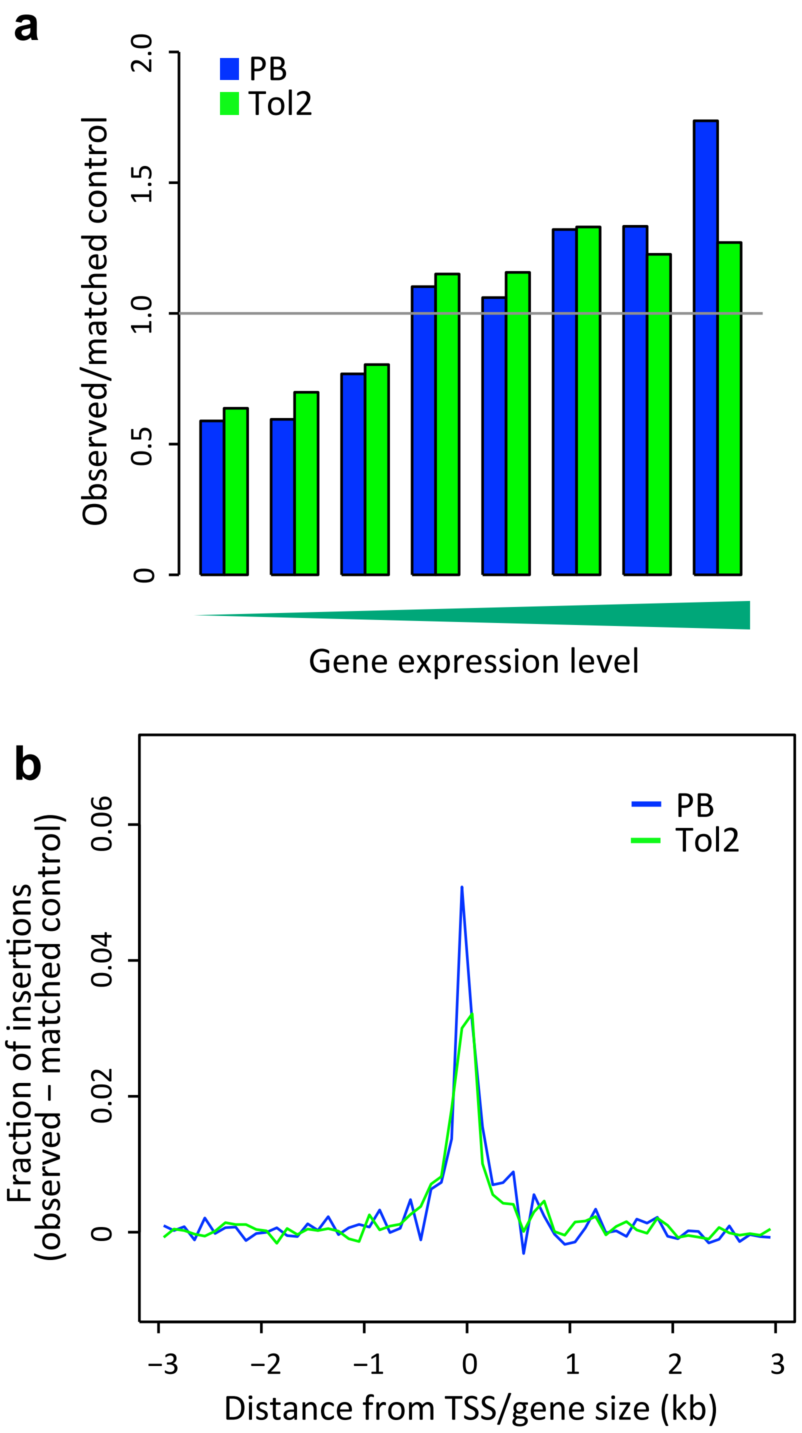

Supplementary Fig. S6. Insertion preference of PB and Tol2 analyzed with Illumina GA2.
(a) Frequency of vector insertion nearby genes (± 50-kb) relative to their expression levels.
(b) Relative distance between TSS and vector insertion sites.
The results of both (a) and (b) were similar to the results of Fig. 3b (left) and 3c that were analyzed with Roche GS FLX using the same lot of genomic DNAs, indicating that the difference of the sequence platform and DNA fragmentation methods (*Hin*dIII digestion or sonication) does not affect our conclusion of insertion site preference.
